# A Program for Solving the Brain Ischemia Problem 

**DOI:** 10.3390/brainsci3020460

**Published:** 2013-04-08

**Authors:** Donald J. DeGracia

**Affiliations:** Department of Physiology, Wayne State University, 4116 Scott Hall, 540 E. Canfield, Detroit, MI 48201, USA; E-Mail: ddegraci@med.wayne.edu; Tel.: +1-313-577-6745; Fax: +1-313-577-5494.

**Keywords:** brain ischemia, neuroprotection, nonlinear dynamics, bistability, cell injury

## Abstract

Our recently described nonlinear dynamical model of cell injury is here applied to the problems of brain ischemia and neuroprotection. We discuss measurement of global brain ischemia injury dynamics by time course analysis. Solutions to proposed experiments are simulated using hypothetical values for the model parameters. The solutions solve the global brain ischemia problem in terms of “master bifurcation diagrams” that show all possible outcomes for arbitrary durations of all lethal cerebral blood flow (CBF) decrements. The global ischemia master bifurcation diagrams: (1) can map to a single focal ischemia insult, and (2) reveal all CBF decrements susceptible to neuroprotection. We simulate measuring a neuroprotectant by time course analysis, which revealed emergent nonlinear effects that set dynamical limits on neuroprotection. Using over-simplified stroke geometry, we calculate a theoretical maximum protection of approximately 50% recovery. We also calculate what is likely to be obtained in practice and obtain 38% recovery; a number close to that often reported in the literature. The hypothetical examples studied here illustrate the use of the nonlinear cell injury model as a fresh avenue of approach that has the potential, not only to solve the brain ischemia problem, but also to advance the technology of neuroprotection.

“In the medical profession there are three kinds of practitioner:

1. Empirics don’t go in for the pursuit of reasons or causes. All they want is empirical facts that will enable them to say “This was helpful (or harmful), so it might be so again in the next case of this sort.”
2. Simple-method physicians attend only to empirical facts which they think they have traced back to reasons or causes.
3. Reasonable physicians who have tried to perfect experience by combining it with the search for causes.”

—G.W. Leibniz [1]

## 1. Introduction

**Figure 1:**
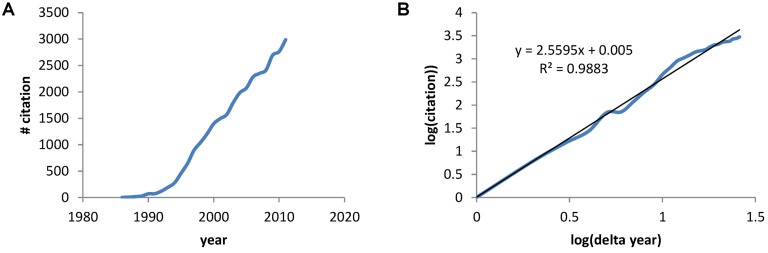
(**A**) The number of papers acquired via PubMed for the search term “neuroprotective” plotted verses the year. (**B**) A log transform of the plot in A where “years” is now expressed as the number of years with 1986 starting as year 1.

The earliest paper on PubMed using the term “neuroprotective” was a 1986 Stroke paper by Silverstein *et al.* showing the calcium antagonist flunarizine protected against hypoxia-ischemia brain damage in immature rats [2]. A PubMed search of the term “neuroprotective” gave 30,815 papers between 1986 and 2011. A plot of the number of papers by year shows the rapid increase in papers (Figure 1A) that, when plotted on a log-log axis reveals a power law increase with exponent 2.6, and a correlation coefficient =0.988 (Figure 1B). Thus, we see a mathematical expression describing the accumulation of papers about neuroprotection over the previous 26 years.

Seidl and Potashkin (2011) defined neuroprotection as: “…aims to prevent or slow disease progression and secondary injuries by halting or at least slowing the loss of neurons” [3]. Yet, in spite of >30,000 papers, it is well-known that almost all neuroprotection clinical trials for focal (stroke) or global (cardiac arrest and resuscitation) ischemic brain injury have failed [4]. Both tPA and hypothermia at present provide relief to only a small percent of patients suffering from ischemic brain damage [5,6]. Debates about the failure of neuroprotection likely account for a significant percentage of the >30,000 papers (e.g., such as [7,8,9,10,11]).

Showing the power law underlying the growth of papers about neuroprotection is unproblematic because it is generally appreciated that information grows rapidly over time. However, the idea that a mathematical expression can be written for the growth of cell damage as a function of the injury magnitude is a new idea that we published in 2012 as a nonlinear dynamical model of cell injury [12]. Here we apply this model specifically to brain ischemia and explore the implications for neuroprotection.

### Overview of the Present Paper

We begin by stating that the present paper presupposes the Reader is familiar with our previous publication of the nonlinear dynamical model of cell injury [12]. Here we aim to achieve the following goals:

1. To theoretically apply the nonlinear dynamical cell injury model to brain ischemia,
2. To serve as a tutorial on the application of pertinent dynamical concepts and methods,
3. To do something that, to our knowledge, is unique in the field: to simulate the expected results of experiments beforehand, illustrating the predictive property of a deductive mathematical framework,
4. To illustrate application of the equation solutions to neuroprotection.

To accomplish these goals, the paper steps through seven major topics that follow the natural order required to operationalize and apply the nonlinear dynamical model of cell injury to brain ischemia:

(1) Since one of our goals is didactic, we must bridge from the current inductive approaches well understood by working experimentalists in the field to a deductive mindset. We therefore elaborate the model focusing on the meaning of the parameters and variables and cast these in light of well-understood empirical features of ischemic brain injury.

(2) The next step discusses how to experimentally measure the cell injury dynamics of brain ischemia. We present one possible experimental protocol that will measure the dynamics of the model variables, *D* and *S*, following global brain ischemia. This step illustrates that, like physicist, we must systematically vary the experimental system, which corresponds to varying equation parameters that we control, so that we can measure those parameters we do not control. We also discuss what will be entailed in measuring *D* and *S*, and provide pilot data that might constitute the first measurement of *S*.

(3) Next, as stated above, we use the mathematical equations to simulate one possible answer to the experimental protocol. This is a **mock simulation**. Its purpose is didactic: to illustrate to experimentalists what we expect our measurements to look like and how the raw data would be analyzed to reveal the cell injury dynamics of our simulated brain ischemia system.

(4) By going through the above exercises, we come to the payoff: we solve our mock simulation of brain ischemia. This is called “fully determining” the equations of the model. The product we obtain from fully determining the model we call “master bifurcation diagrams”. These reveal all possible states of the system.

(5) Having fully solved the mock brain ischemia, we then illustrate what can be done with the solutions by first applying the master bifurcation diagrams to the study of focal brain ischemia. We discuss technical hurdles that must be overcome to measure the model in the context of stroke.

(6) Finally, we come to neuroprotection. The definition above indicates slowing or preventing the death of neurons. Preventing the death of an injured cell on a pro-death trajectory is a process we called “flipping state” because it requires bistable system dynamics [12]. First, we illustrate something unanticipated in the field. Given a fully determined system, **all possible states of neuroprotection can be theoretically calculated beforehand**, **independent of any physical instantiation of the mathematical solutions**. This follows from using a deductive framework and is familiar to physical scientists and engineers, but unfamiliar to the inductive approaches prevalent in current biomedical research. Second, two new calculations are presented, one for global and one for focal brain ischemia, that show the **emergence of nonlinear effects that place strong constraints on the ability of an injured system to flip state**. We identify two such effects: (1) it is sometimes not possible, in principle, to flip state, even if the system is bistable, and (2) sometimes flipping state can be a case of diminishing returns.

(7) We close out the paper considering: (a) the possibility that our equations are incorrect, and (b) how to extend the model to make it more effective for therapeutic application.

Clearly, successful neuroprotection is the end goal of all experimental work on brain ischemia. In addition to the new theoretical discoveries, an important concept emerges in the present work that sheds additional light on the failure to achieve neuroprotection after brain ischemia: **We must clearly distinguish between the science of brain ischemia and the technological application of this science in the form of neuroprotection**. This distinction is presently muddled in the field, where the criterion of success of a scientific study, paper, or grant application is that it must show an improvement in outcome to be considered legitimate. Our results here suggest this mindset is flawed. This tangling of the science and the technology is like expecting physicists studying gravity to produce rockets. Physicists make science, engineers make rockets. The obvious and strong desire to accomplish the end goal of neuroprotection has distracted focus from the pure science, which has helped neither the science nor neuroprotection. Thus our work here clearly demarcates the problem itself, brain ischemia, from one possible technological application to stem from solving that problem, neuroprotection.

As with our previous work in this area [12,13,14,15,16], the present paper is not easy only because it moves in territory unfamiliar to workers in brain ischemia who mostly utilize inductive empirical methods. The present discussion is a necessary stepping stone to making the nonlinear view of cell injury an empirical reality.

## 2. The Nonlinear Dynamical Model of Cell Injury Applied to Brain Ischemia

### 2.1. The Nonlinear Dynamical Model of Cell Injury

Having developed the nonlinear model of cell injury in [12], the simplest form of the model is introduced:

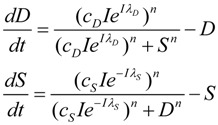
(1)
Where,

*D* is the total amount of damage,
*S* is the total induced stress response,
*c_D_* is the toxicity of the damage agent,
*λ_D_* is the change in toxicity with injury magnitude,
*I* is the injury magnitude,
*c_S_* is the strength of the intrinsic stress response of the given cell type,
*λ_S_* is the change in total induced stress responses with injury magnitude,
*n* is the Hill coefficient (for convenience we arbitrarily set the *n* for *S* and *D* equal).

We elaborate these concepts extensively below. Here we begin with some “housekeeping” statements. Equation (1) is rescaled meaning that it outputs values of *D* and *S* ranging only from 0 to 1 at each value of *I*. In this sense one can think of the solutions to Equation (1) as normalized. Unscaled values for *D* and *S*, which can be thought of as absolute values [12], notated *D'* and *S'*, are obtained with the equations:

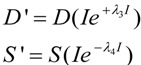
(2)

When *λ*_3_ = *λ*_4_ = 0, the scale factor linking *D* and *S* to *D'* and *S'* is simply *I*. For our subsequent examples, we will use the absolute value output obtained by combining Equations (1) and (2) because changes in absolute values of *D* and *S* are more intuitive to understand. However, there are occasions ahead where normalized solutions present didactic advantages. Since it will be obvious when we are using the normalized or absolute values of *D* and *S*, we will omit usage of the prime.

**Figure 2:**
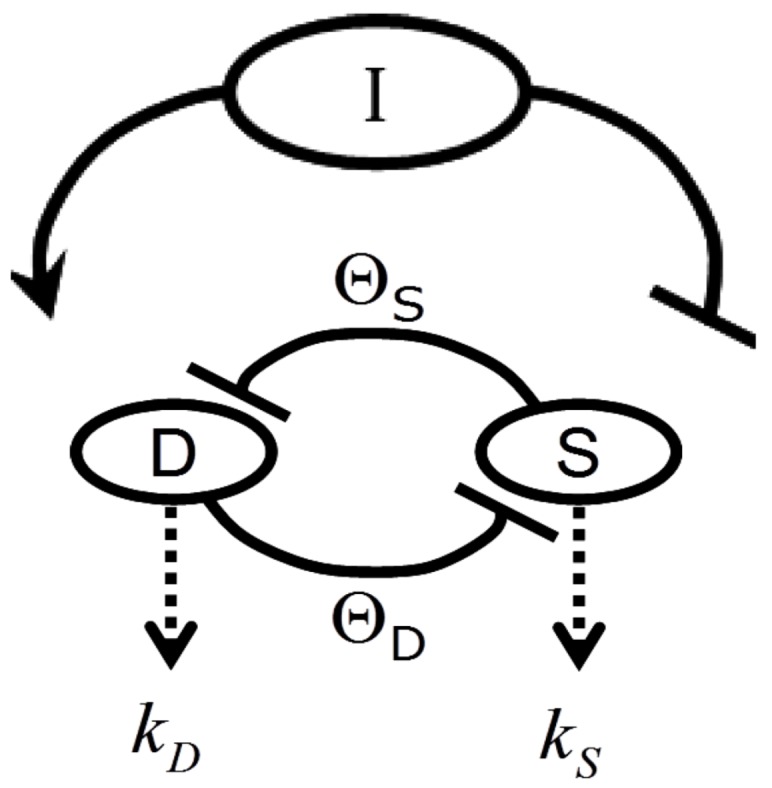
Circuit diagram of the nonlinear dynamical model of cell injury. See text for description.

Figure 2 provides a circuit diagram showing the relationships embodied by Equation (1). At the core of the model is the mutual inhibition of *D* and *S* upon each other. This inhibition is mediated by thresholds of inhibition (*Θ_D_* and *Θ_S_*, defined in the next section). The mutual inhibition is driven by the magnitude of the injury, *I*, which acts as a positive driver on *D*, and a negative driver on *S*. *D* and *S* decay independent of each other as indicated by the decay parameters *k*. As in [12], the decay parameters, as well as velocity parameters *v_S_* and *v_D_* implied by Equation (1), are set equal to 1 in Equation (1), and are not further considered here. The following discussions elaborate on how the variables and parameters of Equation (1) lend themselves to modeling brain ischemia.

### 2.2. Exponential Changes and Injury

As elaborated in [12], *D* and *S* represent the instantaneous sums of all injury-induced molecular damage and stress response pathways, respectively, in an injured cell. The intuition behind the model is that *D* ∞ *e^I^* and *S* ∞ *e*^−*I*^. To realize these proportionalities, we used the Hill equation:

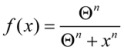
(3)

Equation (3) can be used to define a threshold amount of total damage, *Θ_D_*, which causes a 50% inhibition of *S*. Similarly there is some threshold amount of induced stress responses, *Θ_S_*, which causes a 50% inhibition of *D*. The model assumes that the thresholds *Θ_D_* and *Θ_S_* are not fixed, but instead change with *I*, giving the main expressions behind the model:

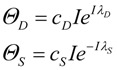
(4)

Thus, *I* does not affect *D* and *S* directly, but instead *Θ_D_* ∞ *e^I^* and *Θ_S_* ∞ *e*^−*I*^. To convert the proportionalities to equations, we need to introduce constants of proportionality and that is why *c_D_*, *c_S_*, *λ_D_* and *λ_S_* enter the picture. We can assign physical meaning to the proportionality constants, which turn out to be critical for applying the model to real-world situations. Further consideration of Equation (4) builds intuition for how to assign interpretations to *c_D_*, *c_S_*, *λ_D_* and *λ_S_*.

### 2.3. Threshold Curves

**Figure 3:**
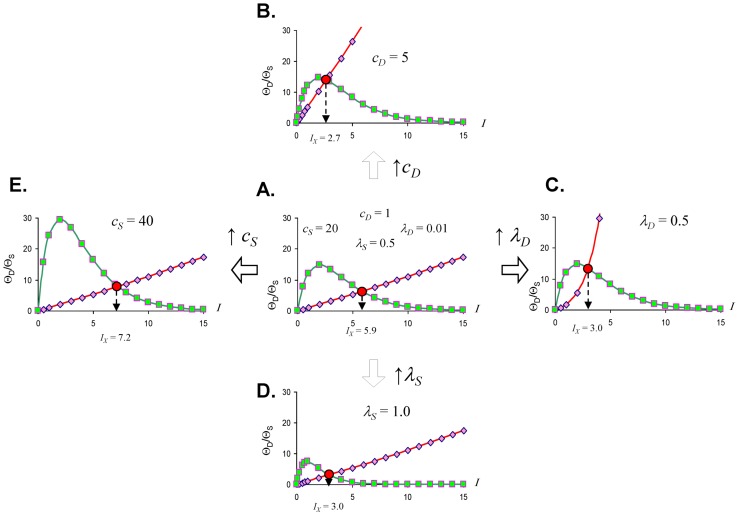
Threshold curves plot *Θ_D_* (red) and *Θ_S_* (green) *versus I*. (**A**) Baseline injury system; (**B**–**E**) increase *c_D_*, *λ_D_*, *λ_S_*, and *c_S_*, respectively, relative to baseline plot in **A**. Parameter values listed on plot.

Studying Equation (4) leads to the idea of an **injury system** consisting of a **cell type**, represented by the *Θ_S_* equation, and a **damage agent** represented by the *Θ_D_* equation. To get a feel for different injury systems, *Θ_D_* and *Θ_S_* are plotted against *I* for fixed values of the parameters *c_D_*, *λ_D_*, *c_S_*, and *λ_S_* (Figure 3). By holding the other parameters fixed, and varying *I*, *I* becomes the control parameter of our analysis. Having *I* on the *x*-axis represents increasing magnitudes of some given *type* of injury. For ischemia, *I* represents the duration of an ischemic insult. For other injuries, *I* will represent the amount of the damage agent such as, for example, increasing concentrations of a toxic substance (e.g., glutamate or AsO_4_^−^), or increasing amounts of applied force in a trauma model. 

In general, *Θ_D_* curves (red) increase with *I* and the *Θ_S_* curves (green) increase to some maximum and then decrease with increasing *I* (Figure 3). Significantly, there is always one (and only one) value of *I* where the *Θ_D_* and *Θ_S_* curves cross, notated as *I_x_*. At *I_x_*, *Θ_D_* = *Θ_S_*. *I_x_* is obtained by setting the *Θ_D_* and *Θ_S_* equations equal and solving for *I*:

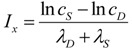
(5)

Equation (5) is one of the most significant features of the nonlinear dynamical model of cell injury. *I_x_* is interpreted as ***the tipping point between life and death*** for an injured system [12]. Intuitively, this is often termed the “cell death threshold”: the amount of injury at which a cell will die. Strictly, *I_x_* is defined as that value of *I* where the fixed point solutions to Equation (1) are equal (the condition D* = S*) [12]. Equation (5) holds for the normalized form of Equation (1), and provides an algebraic means to calculate *I_x_* without solving Equation (1). The importance of Equation (5) is primarily conceptual: it demonstrates that *I_x_*, the tipping point between life and death, the “cell death threshold”, is a function of both the cell type (*c_S_*, *λ_S_*) and the damage agent (*c_D_*, *λ_D_*). This is a critical novel insight of the model in any context of therapy, as illustrated in later sections.

Looking at the effect of individually varying the parameters *c_D_*, *λ_D_*, *c_S_*, and *λ_S_*, we can make generalizations that make intuitive sense. When *c_D_* increases (Figure 3A to 3B), *I_x_* occurs at a lower value of *I*, or, in short, the cell dies easier. Thus, *c_D_* can be interpreted to represent the **toxicity** of the damage agent. When *λ_D_* increases, *I_x_* decreases, and the cell dies easier (Figure 3A to 3C), so *λ_D_* also represents a measure of toxicity. What distinguished *c_D_* from *λ_D_* is that *c_D_* is a multiplier whereas *λ_D_* is in the exponent. If toxic agent A has *c_D_* = 1 and toxic agent B has *c_D_* = 2 (and both have *λ_D_* equal), then B is twice as toxic as A. If toxic agent A has *λ_D_* = 1 and toxic agent B has *λ_D_* = 2 (both with *c_D_* equal), then no simple proportionality holds between the toxicities of A and B, although B will be more toxic than A. Given that *c_D_* is intuitively easier to understand, we focus on *c_D_* in this paper and assume that *λ_D_* is constant in our simulated system ahead. This is done only for simplicity of our examples. Whether it is true in the case of real brain ischemia must be empirically measured and the protocol described ahead would, in principle, measure both *c_D_* and *λ_D_*.

The parameters *c_S_* and *λ_S_* are similarly interpreted. When *c_S_* increase, *I_x_* occurs at a higher value (Figure 3A to 3E), therefore it is harder to kill the cell. Thus, *c_S_*, quantifies how strong a cell is; it quantifies the strength of a cell’s intrinsic stress responses. When *λ_S_* increases, *I_x_* occurs at a lower value and the cell dies easier (Figure 3A to 3D). Therefore 1/*λ_S_* corresponds to a stronger system. It is easier to intuitively assign a meaning to *λ_S_* than to *λ_D_*. Imagine “pounding” on a cell, say with physical force, where, with each hit, the force increases. At some value of force, the cell will collapse. If you have two systems, then the one with the lower value of *λ_S_* will be able to withstand pounding with a higher force. In this sense, *λ_S_* is akin to “stamina” of a sort. The lower is *λ_S_*, the greater the “stamina” of the system to withstand increasing injury magnitudes and survive.

Common sense considerations allow us to put numerical limits on the parameter values. It makes no sense to say that an injury magnitude of zero, or of a negative number, will kill a cell. Thus, *I_x_* must be greater than zero (*I_x_* > 0). As can be seen by Equation (5), to keep *I_x_* > 0, *c_S_* must always be greater than *c_D_* (*c_S_* > *c_D_*), and the sum of *λ_D_* and *λ_S_* has to be greater than zero (*λ_D_* + *λ_S_* > 0). If these constraints are violated, the model is no longer interpretable in terms of cell injury. Similarly, the requirement to multiply by *I* in Equation (4), giving the *Ie*^±*I*^ terms, is to ensure the model outputs 0 when *I* = 0. This too is intuitive: when there is no injury, the dynamics of cell injury must be zero.

### 2.4. Quantifying the Toxicity of Ischemia with c_D_ and λ_D_

With a better understanding of the model parameters, we can apply these to brain ischemia. Elsewhere we lamented the use of the term “ischemia” to describe a quantitative *range* of blood flow decrements [13]. Given our previous work [12], we can now advance a quantitative definition of ischemia that links to Equation (1). Clearly, ischemia is the decrement in cerebral blood flow (CBF), a rate in units of volume/time. For generality, we express CBF as a percent where normal CBF is taken as 100%. Since CBF is a *rate*, we must also consider duration. Ten minutes of 25% CBF is, in some sense, twice the amount of 5 min of 25% CBF. However, based on the extensive work of Hossmann’s lab [17,18], and many others [19,20,21,22], we know that different CBF decrements have different qualitative effects, and therefore are not comparable. A neuron may survive 90% CBF indefinitely, but most neurons will not withstand 30 min of 5% CBF. Is it possible to account for the qualitative effects in a quantitative fashion?

We can imagine a function that equates toxicity to CBF level. While in practice this will require empirical determination, for our purposes here we can mathematically construct such a function. We need a function that is not toxic at close-to-normal CBF, but that becomes progressively more toxic as CBF decreases. A functional form that fits these requirements is the double-exponential Gompertz function (used in the insurance industry to determine mortality rates; [23]). We use the Gompertz form to construct the following expression:

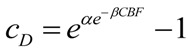
(6)

Here, *c_D_* is exactly as discussed above, *α* and *β* are free parameters, and CBF ranges as a percent from 0 to 100. Solving Equation (6) over the range 0 ≤ CBF ≤ 100 (in percent), with *α* = 0.1 and *β* = 0.75 shows that for CBF from 100% to about 47%, *c_D_* is vanishingly small and can be taken as zero for all practical purposes. *c_D_* increases from 2 × 10^−16^ at 45% CBF to 0.11 at 0% CBF. Thus Equation (6) successfully captures the increasing toxicity of decreasing CBF below a nonlethal cutoff (47%), where the lethal cutoff can be adjusted by the values of *α* and *β*. Our choice of numerical values for *α* and *β* is based on how we shall ahead use the values of *c_D_* just calculated. Let us present a second formula:

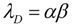
(7)

Thus, *λ_D_* = 0.075. Unlike Equation (6), Equation (7) is not a function of CBF and so, as mentioned above, *λ_D_* will be treated as a constant in the following discussions.

We have transformed the range of CBF decrements into a scale of toxicity quantified by the model parameters that represent the toxic damage agent: *c_D_* and *λ_D_*. Values of (*c_D_*, *λ_D_*) for different values of CBF are shown in Table 1. Thereby, Equations (6) and (7) explicitly account for the qualitative differences in outcome experienced by brain cells subjected to different decrements of CBF.

Again we emphasize: we do not claim Equations (6) and (7) are the correct equations to link CBF decrement to toxicity; only that they have the necessary behavior to fit the requirements of the problem, and work for our purposes here. The advance here is to recognize that **the decrement of CBF**, **which is ischemia**, **will need to be modeled in terms of *c_D_* and *λ_D_***.

**Table 1:** *c_D_* and *λ_D_* calculated by Equations (6) and (7), corresponding to decade decrements in cerebral blood flow (CBF).

CBF (%)	*c_D_*	*D*
**100**	0	0.075
**90**	0	0.075
**80**	0	0.075
**70**	0	0.075
**60**	0	0.075
**50**	0	0.075
**40**	2.0 × 10^−14^	0.075
**30**	1.7 × 10^−11^	0.075
**20**	3.1 × 10^−8^	0.075
**10**	5.5 × 10^−5^	0.075
**0**	0.11	0.075

### 2.5. The Amount of Ischemia as Injury Magnitude, *I*

Even if we now can express different CBF decrements as the pair (*c_D_*, *λ_D_*) we must still account for the duration that the brain tissue experiences a given CBF decrement. The duration for which a brain is exposed to a specific decrement of CBF equates to the **intensity** of exposure to that CBF decrement. In the scope of the model, the amount or intensity of the damage agent is represented by the magnitude of injury, *I* [12]. Thus, assigning numbers to the triplet (*c_D_*, *λ_D_*, *I*) unambiguously defines any duration of any lethal CBF decrement, and we have succeeded in providing a quantitative definition of ischemia for all possible cases, which respects the qualitative differences in outcome.

### 2.6. The Injured Cell as c_S_ and λ_S_

The model abstracts a cell such that only its intrinsic ability to resist being damaged is represented. This is quantified by *c_S_* and *λ_S_* as described above. Here we elaborate on how this logic can be applied to brain ischemia. Consider the following sequence of cell types with respect to their susceptibility to ischemic damage: a CA1 hippocampal pyramidal neuron, a dentate gyrus granule neuron, a brainstem motor neuron, a cardiomyocyte, a hepatocyte, and a skeletal muscle myocyte. The cells types are listed from the most to the least susceptible to ischemic damage, based on empirical observations. Where a CA1 pyramidal neuron will die at some time after exposure to 10 min of 0% CBF [24], a myocyte can withstand over 1 h of 0% CBF [25]. The other cell types fall in graduated degrees between these extremes. Biological factors clearly underpin these differences. For example, myocytes store glycogen and possess a strong creatinine system, both of which allow regeneration of ATP, and are much diminished in neurons [26].

**Figure 4:**
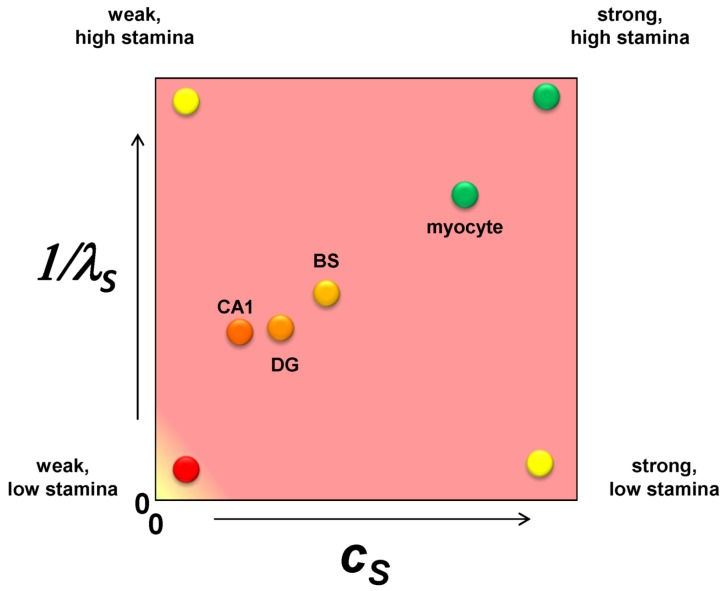
A plot of *c_S_ vs.* 1/*λ_S_* with hypothetical values for a CA1 pyramidal neuron (CA1), a dentate gyrus granule neuron (DG), a brainstem motorneuron (BS) and a skeletal muscle myocyte. The labels at each vertex indicate the qualitative interpretation of the relative parameter values.

It is only one additional step to imagine converting the nonparametric ranking of ischemic susceptibility into specific numerical values. Applying these ideas to brain ischemia, we should consider each cell type in the brain as a point on a plane whose axes are *c_S_* and 1/*λ_S_* (Figure 4). The CA1 pyramidal neuron is the weakest of all the neuron types with respect to ischemic injury so it has the lowest value of *c_S_*. A dentate granule neuron is stronger than a CA1 neuron and will have a higher value of *c_S_*. A brainstem neuron is stronger still and will have a greater value of *c_S_*. However, in comparison to a skeletal muscle myocyte, the three neuron types will have relatively closer values of *c_S_*, and the myocyte will have a value for *c_S_* outside the range of the neuron types. These are illustrated in Figure 4, with similar relative relationships given for 1/*λ_S_*. Because the following examples will focus only on one cell type, both *c_S_* and *λ_S_* will be true constants in the exercises performed in this paper.

## 3. Operationalizing the Nonlinear Dynamical Model of Cell Injury

### 3.1. Preliminary Comments

We now discuss the operational implementation of the theory. The key concept linking theory to practice is the idea of **fully determined**. Fully determined means all of the equation inputs are known so that the equation can be solved. There are two classes of inputs to Equation (1): (1) the parameter values, and (2) the initial conditions.

In dynamical applications, measuring the system parameters is of central importance. Having empirically-determined parameters allows the fully determined model to be solved. Studying the solutions reveals the system’s behavior, predicts how the system will change under various circumstances, and therefore allows rational manipulation of the system. This is the logic with which physical scientists and engineers are familiar, and which our model brings into a biomedical context. Thus, the experiments described below are designed to empirically measure the parameter values so Equation (1) can be fully determined.

There are two main considerations to experimentally measuring the model: (1) the global experimental strategy to measure the parameters, and (2) actually measuring *D* and *S*. We first discuss the global strategy for operationalizing the model, under the assumption we can measure *D* and *S*, without specifying how to do this. Afterward, we discuss measuring *D* and *S* and show that, in principle, it is feasible.

We progress in the following order: (a) the experimental design is rationalized and described, (b) we next simulate data for this experimental design using mock values of the system parameters (those that we would in fact be seeking to measure), (c) next, for didactic purposes, we **pretend** we do not know the parameter values and we analyze the simulated mock data to recover the parameter values using the appropriate methods, and finally, (d) we use the parameters obtained from the experimental design to show what the fully determined solution looks like in the form of master bifurcation diagrams.

### 3.2. Measuring the Model Parameters for Global Ischemia

The analytical methods for measuring the model parameters fall in the scope of **time series analysis**. Time series analysis constitutes a diverse array of methods to analyze time varying data, ranging from classical signal processing methods (e.g., Fourier analysis) to newer methods to detect chaotic dynamical systems. These methods are widely used in the physical sciences and engineering as a tool of **system identification**, in which dynamical models are built from the measured data. Explaining the various methods of time series analysis is much beyond the scope of this article, and many sources of information are available [27,28,29,30,31,32].

Instead, we illustrate methods applicable to solving Equation (1) in the context of experimental designs any brain ischemia worker will recognize. The experimental data will consist of discreet time points sampled from *D* and *S* time courses following various conditions of global ischemia and reperfusion. The discreet time points are used to reconstruct the *D* and *S* time courses. The *D* and *S* time courses are then curve fit to obtain the system parameters.

Before proceeding to more detailed considerations, it is useful to summarize the main point of this section is by analogy. Everyone knows the formula for a straight line: *y = mx + b*. Here, *x* is the independent variable, *y* the dependent variable, and *m* and *b* are the parameters. In an experiment, *x* might represent a drug concentration that you the experimenter control; *y*, for example, might be the level of phosphorylation of a protein in response to the drug that you, the experimenter, measure. Assume the data is linear. The data is entered into suitable software and fit to the equation of the line. The parameters *m* and *b* are **not** measured; they are calculated automatically by the linear regression procedure. In addition, the fit procedure gives a statistic, the linear correlation coefficient, which tells the statistical confidence for how well the data fit the equation of a line.

To measure the nonlinear model, we follow analogous steps. In brief, one measures *D* and *S* time courses and then curve fits these, using nonlinear regression, to the *D* and *S* time courses output by Equation (1). Through the curve fitting procedure, the parameters are output from the nonlinear regression analogous to how *m* and *b* are output from a linear regression. The nonlinear fit also provides statistics for how well Equation (1) fits the measured *D* and *S* time courses.

### 3.3. Initial Conditions of *D* and *S* Time Courses

Since we need to fit experimentally measured *D* and *S* time course to *D* and *S* time courses output from Equation (1), we will need to solve Equation (1) to obtain the mathematical time courses. Obtaining specific time courses from Equation (1) requires input of specific numerical parameter values and initial conditions [12]. Initial conditions, notated (*D*_0_, *S*_0_), are the numerical values of *D* and *S* at time zero. We experimentally control initial conditions. If we induce ischemia in an uninjured animal, then, at the start of the experiment, the animal has no damage and no activated stress responses; that is, (*D*_0_, *S*_0_) = (0, 0). In reality, there will always be some very small level of endogenous damage and stress responses in an experimental animal. But endogenous *D* and *S* are expected to be very small compared to injury-induced *D* and *S,* and can be approximated as zero for all practical purposes.

In the scope of the current model, initial conditions are intimately linked to therapeutics and the technology of neuroprotection, which is extensively discussed in later sections. We want the simplest case for experimentally measuring the system parameters in the design below. Therefore, we set (*D*_0_, *S*_0_) = (0, 0) by performing the experiments on uninjured animals with no other treatment than the injury conditions.

### 3.4. How Equation (1) Parameters Relate to the Protocol for *D* and *S* Time Course Sampling

*D* and *S* are the dependent variables of our dynamical model. To measure the system parameters, we need to measure how *D* and *S* change with the independent variable time, *t*, while systematically changing injury conditions that correspond to the equation parameters. Summarizing the interpretation of Equation (1) parameters in terms of brain ischemia:

*I* = the duration of ischemia (e.g., the duration of a specific CBF decrement),
(*c_D_*, *λ_D_*) = the toxicity of a specific CBF decrement,
(*c_S_*, *λ_S_*) = the stress response quantifiers of a single cell type,
*n* = the Hill coefficient.

When looking at this list we must ask: which of these parameters do we control and which do we not control? For which do we know the numerical values, and for which not? We clearly control the duration of a given decrement of CBF, which is *I*, and since *I* is duration, we know its numerical value. We also can, in principle, control the CBF decrement by controlling the global amount of blood entering the brain, even if we do not know the numerical values of *c_D_* and *λ_D_* corresponding to the global CBF decrements. We can also control which cell type we evaluate, again, even if we do not know that cell type’s values of *c_S_* and *λ_S_*. We certainly neither control nor *a priori* know the value for *n* in Equation (1). Again, at this point in the discussion, we simply assume we can measure *D* and *S*. 

**Figure 5:**
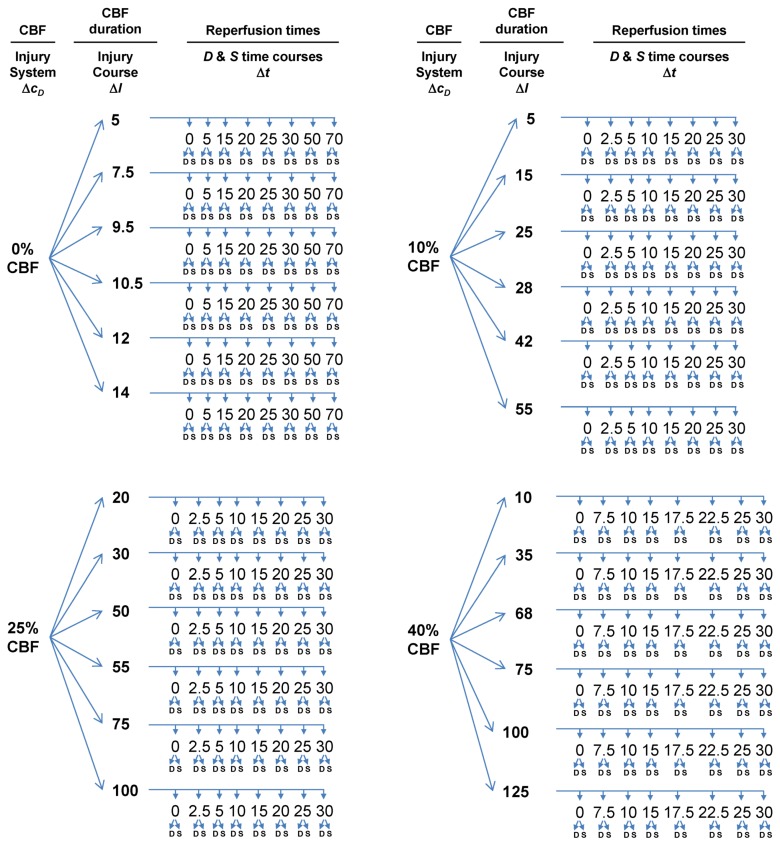
Flow diagram outlining experimental design to measure global brain ischemia injury dynamics at lethal CBF values of 0%, 10%, 25% and 40%, each corresponding to a different value of *c_D_*. For each CBF decrement, multiple *I* values are sampled, giving injury courses. Finally, individual time courses are measured at each *c_D_* and *I*. The numbers used do not have real units because these are hypothetical values for the sake of example. Δ*c_D_*, Δ*I*, and Δt indicate which parameter/variable is being controlled. Finally, for each time point where *D* and *S* are sampled, the sample occurs from the same neuron populations. Therefore, with this protocol, *c_S_* and *λ_S_* are maintained constant.

Because we can control: (a) the global CBF level, (b) the duration of any given CBF level, and (c) the cell type we sample from, we can design an experimental protocol that will allow us to measure *D* and *S* time courses by varying (a) and (b) and keeping (c) constant. This will result in a large number of measured *D* and *S* time courses from the same cell type. From these time courses, the model parameters can be estimated by nonlinear curve fitting to Equation (1). The following protocol turns out to be relatively straightforward in its design, but because we must measure the system while systematically varying parameters, the protocol is tedious, repetitious and laborious (Figure 5).

The main experimental parameter we will control is the global CBF level in the animal’s brain, again corresponding to (*c_D_*, *λ_D_*). As described above, we assume, via Equations (6) and (7) that *c_D_* will vary with CBF, but that *λ_D_* will be constant across CBF decrements. We require a suitable animal model of global brain ischemia. Our lab routinely uses bilateral carotid artery occlusion with hypovolemic hypotension in rat [33]. For our mock example, we’ll assume the use of this animal model. Normally, this model is performed such that the ischemia is 0% CBF, or global complete forebrain ischemia. In the following example, we assume, without elaborating here, that this animal model can be modified to allow the experimenter to set the global brain CBF at any decrement from 100% CBF to 0% CBF. From here out we use the terms “ischemia” and “CBF decrement” interchangeably.

Next, we need to sample a specific cell type in the brain after ischemia and reperfusion. For our mock experimental design, we imagine microdissecting hippocampal CA1 and that our measurements of *D* and *S* reflect the pyramidal neurons of CA1. While there are obvious technical caveats here (e.g., the multiple cell types in the dissected tissue), these are not lethal to our arguments, so we ignore such technical details for the sake of keeping the mock exercise as simple as possible.

To set up the time course analysis we need to systematically vary global CBF as follows:

1. Choose multiple lethal CBF decrements.
2. At each CBF decrement, choose multiple ischemia durations.
3. At each ischemia duration choose multiple time points to sample the reperfusion time course.
4. At each sampled time point, measure *D* and *S*.

With this design, we will solve the model for one specific brain cell type, the CA1 pyramidal neuron. We can see that *c_S_* and *λ_S_* are true constants in this set up. We now provide additional commentary about each step.

#### 3.4.1. Multiple CBF Levels = Injury Systems

We set the α and β parameters of Equation (6) such that CBF > 47% is not lethal and CBF ≤ 47% is lethal. The real cutoff would need to be experimentally determined, but for the sake of our mock exercise, we take 47% CBF to be the cut-off. Because CBF is a continuous variable, an ideal experimental design might be to sample every 1% step of CBF from 47% to 0% (e.g., 47%, 46%, 45% ... 0%). However, this would be unwieldy in practice, and redundant in terms of the information produced. Instead, we design the experiment to sample from the maximum lethal CBF, 0% CBF, and a minimum lethal CBF of 40% CBF, close to the 47% cutoff (We work with 40% CBF, because, as seen in Table 1, the corresponding *c_D_* = 2 × 10^−14^; to use much smaller numbers runs into the inherent limits of double precision on modern computers). In addition, two values of CBF between 0% and 40%, e.g., 10% CBF and 25% CBF are chosen. Thus, we sample only four CBF decrements across almost the whole hypothetical lethal CBF range calculated by Equation (6). The four CBF data points can provide the minimum information that will allow us to reconstruct the mathematical relationship between % CBF and *c_D_* given by Equation (6). Because each CBF decrement has a unique value for *c_D_* (Table 1), this step sets up four different **injury systems** for measurement: 0%, 10%, 25% and 40% CBF.

#### 3.4.2. Multiple Ischemia Durations = Injury Courses

At a given % CBF, some ischemia durations are lethal but others are not. Consider complete global brain ischemia, 0% CBF. It is well-known that 10 min of 0% CBF is lethal to rat CA1 neurons. However, 2 min 0% CBF is not lethal. Thus, for any lethal level of CBF, there will be some exact ischemia duration that is the tipping point between lethal and nonlethal for a given cell type in a given species. This tipping point is *I_x_* in our model. For the time course analysis to work, we need to sample on both sides of *I_x_*. For rat CA1 neurons, we assume the tipping point, *I_x_*, is 10 min of 0% CBF. Therefore, for 0% CBF, a decent sample of ischemic durations is: *I* = 5, 7.5, 9.5, 10.5, 12, and 14 min of 0% CBF, giving us three ischemia durations on either side of *I_x_*, two of which are close to *I_x_* = 10 min ischemia. Explained above, ischemia duration is the parameter *I*, and thus serves as the *control* (*or order*) *parameter* for each injury system. We call a series of time courses generated using the same injury system at different values of *I* an **injury course** [12]. 

*I_x_* will not be the same for all four CBF levels. That is, 10 min of 40% CBF will not kill CA1 neurons; they will require some longer duration of 40% CBF before they die. Therefore, the ischemia durations, e.g., the parameter *I*, sampled for each CBF level will different. In real life, because these are not routinely studied, pilot studies would first need to establish the tipping point (=*I_x_*) durations of ischemia at 10%, 25% and 40% CBF. For time course analysis, ischemia durations on either side of *I_x_* need to be sampled. We calculated beforehand appropriate choices of ischemia durations for the 10%, 25% and 45% CBF levels used in our mock experiment (Figure 5).

#### 3.4.3. Reperfusion Time Courses

Once we have chosen the durations of global ischemia (e.g., *I*) to use at each level of CBF, we measure *D* and *S* over reperfusion time courses. There are two cases that set the total reperfusion duration over which *D* and *S* must be measured [12], either: (1) the duration of recovery: which is the duration it takes for the cell of interest to revert to the pre-injury state, or (2) the duration to cell death: the time it takes for the cell of interest to completely disintegrate due to death. Therefore, suitable sampling at different times of reperfusion, until either conditions 1 or 2 are met, is required. We have pre-calculated suitable reperfusion sampling times for our mock experiment (Figure 5).

### 3.5. The Simulation

To carry out this exercise we used Equations (1) and (2) to simulate the data for the experimental design in Figure 5. The numerical values of *c_D_* and *λ_D_* were calculated by Equations (6) and (7). It was next calculated what values of *c_S_* and *λ_S_* would cause *I_x_* = 10 for the 0% CBF case, thereby simulating CA1 neuron death after 10 min of complete global brain ischemia. This provided the values (*c_S_*, *λ_S_*) used in all other cases. We emphasize that all numbers used in this example are arbitrary. To actually execute the design described here, conventions and units need to be adopted that would affect any real numerical values used to solve Equations (1) and (2). The parameter values of our simulated injury systems are shown in Table 2. Via Equation (5), *I_x_* was also calculated in Table 2.

**Table 2:** Parameter values for simulated *D* and *S* time courses. The Hill coefficient *n* = 4.

CBF (%)	*c_D_*	*λ_D_*	*c_S_*	*λ_S_*	*I_x_*
**0**	0.1052	0.075	10	0.38	10.0
**10**	5.5 × 10^−5^	0.075	10	0.38	26.6
**25**	7.2 × 10^−10^	0.075	10	0.38	51.3
**40**	2.2 × 10^−14^	0.075	10	0.38	76.6

Again we remind the Reader that the aim of a real experiment like that in Figure 5 would be to empirically determine the parameters values shown in Table 2. For our mock simulation, we now pretend we do not know these numbers. Instead, we have set up the experiment in Figure 5, and from it we obtain *D* and *S* time courses. We now illustrate what the data would look like, and how it would be analyzed to recover (or discover in the case of a real experiment) the parameter values.

## 4. The Simulated Experimental Dataset and Its Analysis

### 4.1. The Time Series Dataset

**Figure 6:**
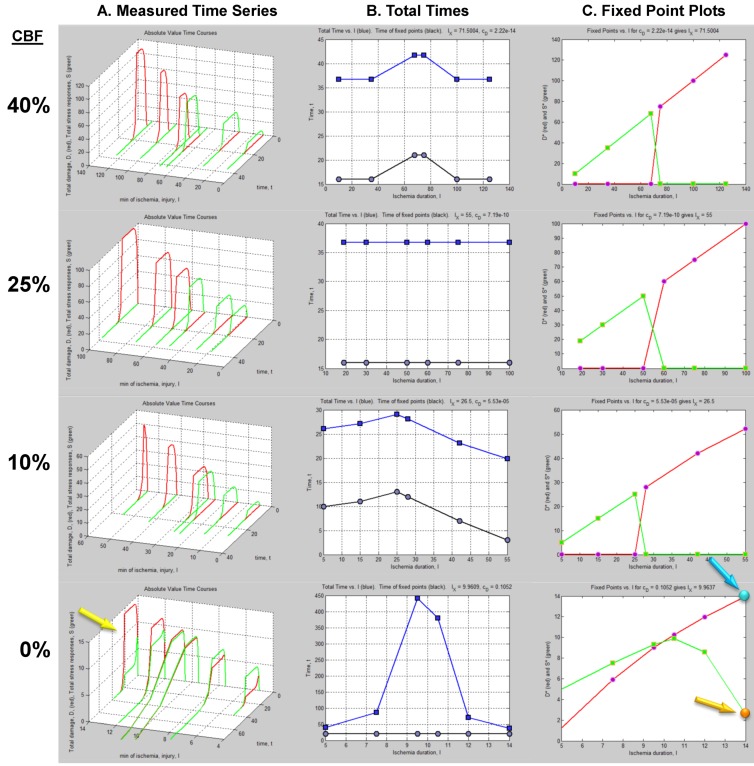
Hypothetical time series “experimental data”. (**A**) Hypothetical measured *D* (red) and *S* (green) time courses at %CBF as indicated. The *x*-axis is the parameter *I*, here the ischemia duration; *y*-axis is time, *t*, and *z*-axis is *D* or *S*. (**B**) Total duration of *D* and *S* time courses (blue lines) and durations to the fixed points (black curves). (**C**) Fixed point plots of D* (red) *vs. I* and S* (green) *vs. I*. Yellow arrow indicates time courses used for derivative in Figure 7. Blue and orange arrows point to D* and S* calculated by derivative plot in Figure 7.

The column of graphs in Figure 6A shows the simulated time courses from the experimental design in Figure 5. Shown are continuous *D* and *S* curves that would result from connecting the discreet sampled time points. *D* and *S* time courses are always plotted in red and green, respectively. The time course data illustrate that each CBF decrement has its own injury dynamics. For the 0% CBF case, there is a gradual increase in peak *D* with *I*, and peak values of *S* also gradually increase and then decrease. However, in the 10%, 25% and 40% cases, gradual changes in *D* and *S* with *I* are absent. Instead, *D* is very close to zero early in the injury courses and then abruptly increases at higher values of injury, increasing gradually thereafter. Similarly, *S* gradually increases in the early parts of the injury courses, but then abruptly drops to near zero at higher injury values. Such abrupt changes are characteristic of bistability and indicate bifurcations occurring across the injury courses, the implications of which are explored ahead. 

Another view of the time series data is provided by plotting the total duration of each time course *vs. I* at each CBF level (Figure 6B, middle columns, blue curves). Here, the 40% CBF shows maximal duration of ~42 time units around *I* = 70. For 25% CBF, the total time curve is flat across the injury course, meaning it took the same duration of ~37 time units for the cells to either recover or die. For 10% CBF, the total time increases to about 30 time units at *I* = 25, and declines after. For 0% CBF, total times are ~450 time units around *I* = 10, 10 times greater than the other CBF levels. Thus, our choice of parameters ended up simulating the well-known delayed neuronal death of hippocampal CA1 neurons following 10 min complete global brain ischemia [24]. However, given the arbitrary nature of the numerical parameter values, this is a fortuitous coincidence for our example data.

### 4.2. Fixed Point Diagrams

We now discuss how the raw time course data is analyzed to determine the **fixed points**, also called **attractors**, for each individual time course. By definition, a fixed point/attractor is where all variables of the system have a **rate** equal to zero **at the same time** [30]. In our case this means *dS*/*dt* = 0 at the same point in time that *dD*/*dt* = 0. The point *D* where *dD*/*dt* = 0 is notated D*, and similarly for S*. Thus, to determine (D*, S*), one takes the derivatives of the *D* and *S* time courses and determines when both derivatives equal 0.

In practice, one could either curve fit the time courses and differentiate the fit equation, or the attractors can be estimated directly from the time series data by subtracting sequential values of *D* or *S* across the time series to give values of delta *D* (Δ*D*) or delta *S* (Δ*S*), e.g., as in:

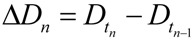
(8)

**Figure 7:**
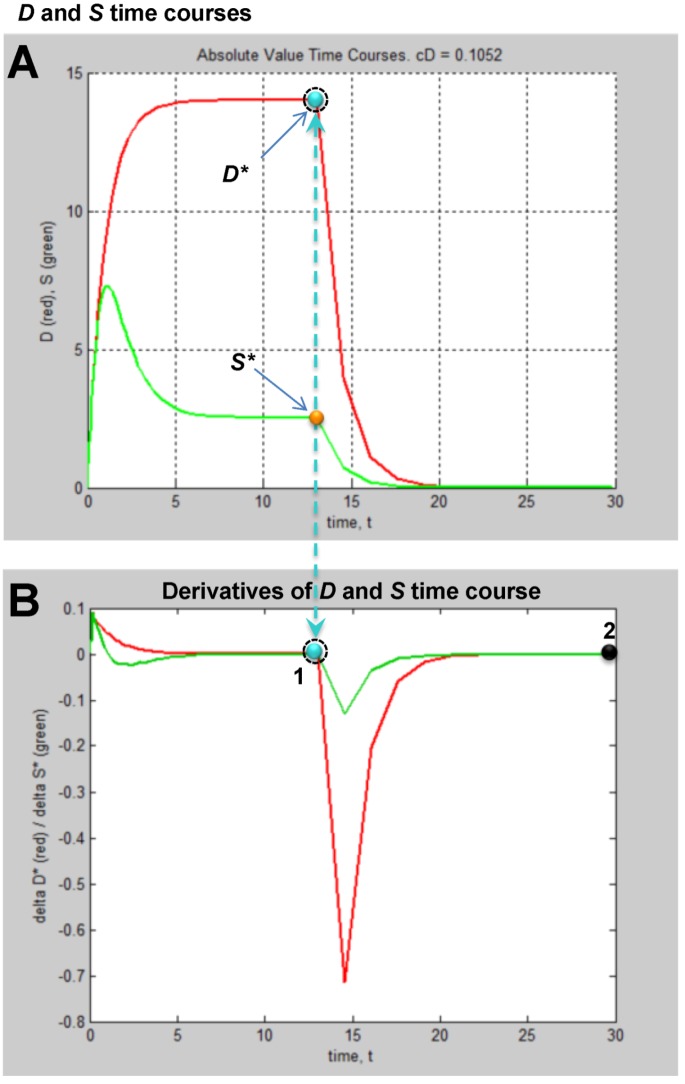
Example analysis to determine fixed points from *D* and *S* time courses. (**A**) *D* (red) and *S* (green) time courses marked on Figure 6. (**B**) Derivative plots of *D* (red) and *S* (green) time courses in panel A. Blue dashed line links the corresponding attractors on time courses to point 1 that indicates where *dD*/*dt* = *dS*/*dt* = 0. At point 2, *dD*/*dt* = *dS*/*dt* = 0 because it is the end of the time courses.

Then one determines the values of Δ*D* and Δ*S* that are both closest to zero at the same time. This procedure is illustrated in Figure 7. Figure 7A shows the *D* and *S* time courses for the 0% CBF case at *I* = 14 min of ischemia (this pair of *D* and *S* time courses is marked by the yellow arrow in Figure 6). In this time course pair, *D* dominates *S*, and the cell disintegrates completely after 20 time units. 

Figure 7B shows plots of Δ*D* and Δ*S vs.* time, *t*, and was constructed by taking the difference between sequential values of *D* and *S* from the time courses, as indicated by Equation (8). There are two points in time where the derivative curves equal 0, marked “1” and “2” on the derivative plot. The point marked 2 merely represents the end of the *D* and *S* time courses. It is point “1” that is of interest. The blue dashed line connects this to the corresponding points on the *D* and *S* time course, intersecting the attractor points D* and S*. Thus, for the *D* and *S* time courses at *I* = 14 and 0% CFB, the attractor state is (D*, S*) = (14, 2.5) (the points D* and S* are marked by arrows on Figure 6 as blue and orange points, respectively, in column C).

(D*, S*) can be similarly determined for all of the other time courses in the time series. Then, plots of D* vs. I and S* vs. I give **fixed point plots** for each injury system (Figure 6C). The time, *t*, at which the fixed points occur is plotted *vs. I* (black curves, Figure 6B). 

The patterns of gradual or abrupt change seen in the time series data are also present in the fixed point plots. Gradual changes of D* and S* are seen in the 0% CBF plot, while the others show abrupt changes in the fixed points from zero to some other value. 

Significantly, similar to the threshold curves (Figure 3), there is one specific value of *I* where the D* vs. I curve crosses the S* vs. I curve. This value of *I* is *I_x_*, the tipping point between life and death. The curves in Figure 6C illustrate the formal definition of *I_x_* as the value of *I* where D* = S*.

Another feature of the fixed point plots requires mention by way of warning. In the 10%, 25% and 40% injury courses, both S* and D* increase linearly over sub-ranges of *I*. For the 0% CBF case, D* also approximates a straight line, and could be adequately fit by linear regression. But this data was obviously generated from the nonlinear differential Equation (1). Thus, this example stands as a warning that even though data can superficially appear linear, it nonetheless may derive from an underlying nonlinear theory. 

### 4.3. Parameter Estimation from the Dataset

We are now at the turning point of our example. The plots in Figure 6 display the dynamics of our hypothetical global cerebral ischemia and from these the parameters are determined. Given Equation (1), the specific time courses in Figure 6A, and fixed point plots of Figure 6C, there are only specific parameter values that, when plugged into Equation (1) can produce the measured patterns.

We do not discuss the details of the nonlinear fit equation but only briefly describe it. Data points are fit by a least squares regression method such as the Levenberg-Marquardt least squares curve fitting algorithm [34]. This numerical algorithm is used to minimize nonlinear functions over the parameter space of the function. It finds parameters, *p*, that minimizes the sum of the squares of the difference between measured and predicted data points in nonlinear curves.

In addition to determining parameters, the nonlinear regression can estimate the goodness of fit of the data with the model. To do so, one adds a random parameter to Equation (1) whose mean is, by definition zero, and has standard deviation σ [30]. The standard deviation can then be calculated to provide the confidence limits that Equation (1) describes the time course data. The goodness of fit between the empirical data and equation output is often expressed as “*x*-sigma”, as in physics where a 5-sigma criterion of acceptance is used. It is common to use 2-sigma, or *p* < 0.05 in biomedical research; a 5-sigma criteria means *p* < 3 × 10^−7^. 

Thus, given Equations (1) and (2) and data portrayed in Figure 6A, and using these analytical tools, we can estimate the numerical values of the parameters *c_D_*, *λ_D_*, for the CBF decrements, and determine *c_S_* and *λ_S_* for the CA1 pyramidal neuron. Importantly, the design in Figure 5 has a self-consistency check built into it: We can check the empirically derived parameters via Equation (5): when plugged into Equation (5), the empirical parameters must give the measured values of *I_x_*.

Finally, not shown in the design of Figure 5, but well understood by experimentalists, one must include multiple animals at each time point sampled to generate averages for the measured levels of *D* and *S*. This in turn causes the parameters to be expressed as means with some variance. Knowing the range of the parameters within some statistical limit is the best that can be achieved in any event, whether one is doing physics or biomedical research. We now discuss how the successful completion of a design like that described above offers a complete solution to the global ischemia problem.

## 5. Solving Global Brain Ischemia

**Figure 8:**
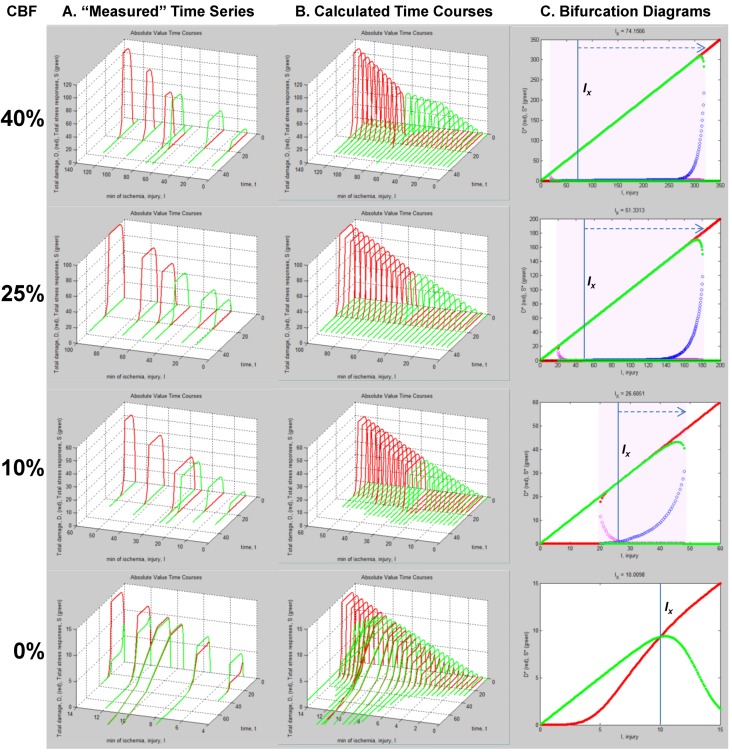
Plots calculated from fully determined model. (**A**) Repeats hypothetical time series experimental data from Figure 5. (**B**) Using Equations (1) and (2) to calculate intervening time courses that were not experimentally measured. (**C**) Using Equations (1) and (2) to calculate bifurcation diagrams that reveal if and where the system undergoes bistable phase transitions. Purple areas mark bistable regions. *I_x_* is indicated by vertical line on each bifurcation diagram. Dashed arrows mark therapeutic region on each injury course.

With experimentally determined parameters the model is fully determined, and the parameters can be plugged into the model equations. How this would look is shown in Figure 8B,C. Here we repeat the simulated “empirical” data in column A now adjacent to plots generated by Equation (1), filling in the missing data from the time series. We can now see in full how the *D* and *S* time courses appear at all values of *I* for a given % CBF (Figure 8B).

The pay-off for being able to plug empirically-determined parameters into the model equations is we can now ***calculate*** complete bifurcation diagrams. Comparing columns C between Figure 6, Figure 8, there is new information in the equation-generated bifurcation diagrams. These now show whether or not the system undergoes **bifurcations** from **monostable** to **bistable** phase planes, and precisely where this occurs on the injury course. The bistable regions of the equation solutions are shown by the purple rectangles, which mark the range of *I* values where the system is bistable. The magnitudes of the bistable regions are listed in Table 3. In our mock example there was no bistability in the 0% CBF case, however the range of bistable *I* values increased with % CBF. Bistability is intimately linked to therapeutic application of the model, discussed ahead.

**Table 3:** Ranges of bistability for the four measured injury systems.

CBF (%)	bistable start	bistable end	bistable I range
0	n/a	n/a	0
10	18.4	53.8	35.4
25	18.4	187.2	168.8
40	18.4	318.6	300.2

### Master Bifurcation Diagrams

With a fully determined model, we can now calculate ***all*** bifurcation diagrams across a continuous range of any of the parameters. In the present example, *I* and *c_D_* were varied. Therefore, we can calculate the bifurcation diagrams across continuous ranges of both. Since the dynamics of our system, a hypothetical global ischemia applied to a hypothetical CA1 neuron, is defined over ranges of *c_D_* and *I*, this calculation ***solves the system for every possible state***.

Since *c_D_* ranged from ~10^−14^ at 40% CBF to ~0.11 at 0% CBF (Table 1), we can calculate all bifurcation diagrams over this range. This amounts to stacking the individual bifurcation diagrams (e.g., as in Figure 8C) along a third axis, the value of *c_D_*, and filling in by calculation the remaining bifurcation diagrams (analogous to how missing time courses were calculated in Figure 8B).

These calculations produce what we call “master bifurcation diagrams” showing every possible state of our hypothetical global cerebral ischemia with respect to our hypothetical CA1 neuron. The master bifurcation diagram consists of 2-dimensional surfaces of D* and S* values in a 3D space whose axes are injury, *I*, *c_D_*, and D* or S*.

**Figure 9:**
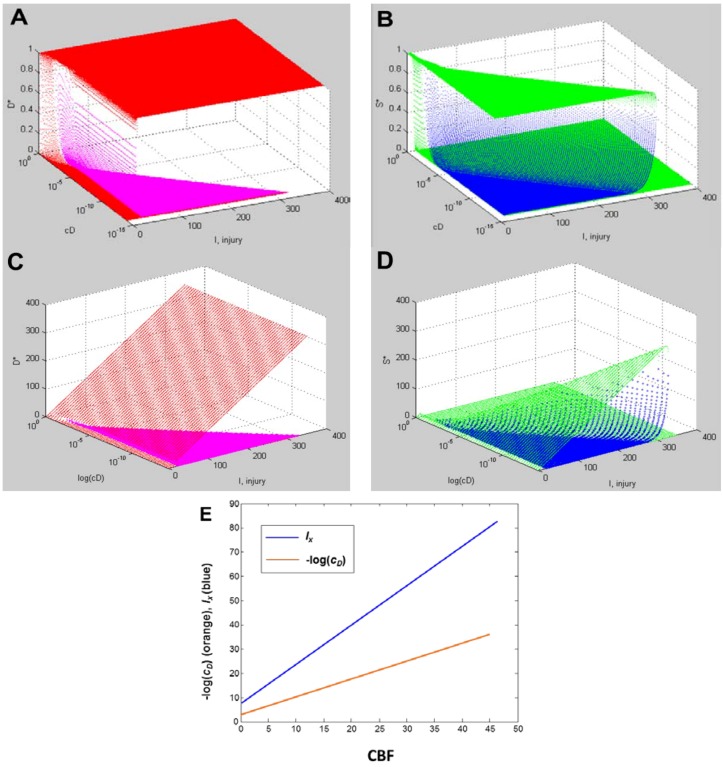
Master bifurcation diagrams. Normalized D* (**A**) and S* (**B**) plots *vs. I* and *c_D_*. Absolute value D* (**C**) and S* (**D**) plots *vs. I* and *c_D_*. In **A**–**D**, *c_D_* is plotted as log(*c_D_*). Attractor points are in red (D*) and green (S*) and repeller points are in magenta (D*) and blue (S*).(**E**) Plots of CBF *vs.* −log(*c_D_*) (orange line) and CBF *vs. I_x_* (blue line). Axes as labeled.

Figure 9A,B, and Figure 9C,D show, respectively, the normalized and absolute values master bifurcation diagrams for D* and S*. The log of *c_D_* is plotted since *c_D_* varies over 14 orders of magnitude. The attractors are in red and green, and the repeller are plotted in magenta and blue, for D* and S*, respectively.

In Figure 9A,B, there is a triangular “floor” of repeller points that extend for most of the range of *c_D_*, ending at *c_D_* ~ 0.01. The D* repellers makes a “wall” that runs parallel to the *c_D_* axis. The corresponding repellers for S* also make a “wall” of points following the hypotenuse of the triangular contour, ending at the same value of *c_D_* ~ 0.01. Although not obvious on inspection, the absolute value bifurcation surfaces (Figure 9C,D) have exactly the same topology as the normalized curves.

Equation (6) links CBF to *c_D_*. Figure 9E plots CBF against the negative log of *c_D_*, giving a straight line. Importantly, the bifurcation diagrams give the value of *I_x_* at each *c_D_*. Thus, another way to express the total system dynamics is to plot CBF *vs. I_x_*, which also gives a linear relationship.

This is a truly profound result. These plots reveal all possible solutions of our problem, a hypothetical global brain ischemia, at all levels of lethal CBF decrements, across a range of 350 durations of CBF, for a hypothetical CA1 neuron. The entire system stands transparent and fully revealed. With the master bifurcation diagrams, we can predict, within the statistical error of the empirically-determined parameters, the outcome, whether the system will live or die, for a given duration of a specific level of CBF. We can calculate every possible *D* and *S* time course of recovery or death. Importantly for therapeutic purposes, we can determine if the system is bistable or monostable, providing the information needed to either reverse or slow the injury trajectory.

This closes out discussion of the global strategy to measure the model parameters. We hope to have shown how empirically realizing what has been hypothetically described here has the potential to solve the problem of global brain ischemia.

## 6. Measuring *D* and *S*

### 6.1. General Considerations

The only aspect of the experiment described above that is not possible to perform at present is measurement of the total amount of damage, *D*, and the total induced stress responses, *S*. We elsewhere discussed the rationale and necessity for considering the concepts of *D* and *S* [12,13,14,15,16]. The main point is that, in brain ischemia, as with many other injury modalities, it is now amply understood that the injuries induce many forms of cell damage and many stress responses. Our dynamical cell injury model theorizes that these many molecular pathways are not independent but change in tandem. The most sensible interpretation of Equation (1) is that it describes precisely how all of the molecular pathways of damage and stress response change in a coordinated fashion through time.

The philosophical implication of our model, stated by Leibniz above, is that when we measure products of cellular damage after ischemia we need to dissociate these from ideas of *causation*. No specific damage product, by itself, causes the cell to die. The accumulation of cell damage products is just a consequence of the fact that the cell was injured. The reason, the *cause*, of why a cell dies when it is injured is because it is too weak to overcome the total amount of damage induced by a lethal injury magnitude (this is a qualitative way to say D* > S*). Thus, instead of being causal, the damage products serve as markers of the injury magnitude. From this view, it is the relative amounts of damage products and stress responses that become more important than a qualitative list of either. Thus, the first insight into measuring *D* and *S* is that they are essentially quantitative and not qualitative.

Next, the idea that damage products and stress responses quantitatively change *in tandem* brings out a central concept behind measuring them: **they covary**. We do not use the term “covary” in a statistical sense of correlation, but in a deterministic sense. As can be seen in Equation (1), both *D* and *S* are deterministically locked together, and each is locked to injury magnitude, *I*. For example, given a stronger insult compared to a weaker insult, we are likely to find *more* free radical products, *more* degraded lipids and proteins, *more* protein aggregates, more heat shock response, more anti-oxidant response. That is, since all damage and stress responses change in tandem with injury magnitude, the change in any individual damage product or stress response will contain much the same information as the others, which is the essence of what we mean by “covary”.

There is an important scientific concept that has inspired our conception of *D* and *S*: *temperature*. Temperature is an aggregate measure; it measures the *average* kinetic energy of mols of atoms and molecules. Some atoms are much higher, some much lower than the average, but this variation matters only in its contribution to the average. When we measure the change in temperature, we measure the covariance of the *range* of kinetic energies of the microscopic components. For the purposes to which we put measures of temperature, it would be unproductive to seek to measure the kinetic energy of every atom and molecule. Furthermore, the individual identities of the atoms and molecules are not relevant: 37 °C water is exactly the same temperature as 37 °C air. 

*D* and *S* are to be treated like the concept of temperature. They represent aggregate effects where the qualitative details of the microscopic individual entities are inconsequential except in how they contribute to the aggregate. Elsewhere we discussed the relationship between the individual molecular pathways and how they act in tandem as a network [16], and so do not pursue that issue here. For all practical purposes, we can think of *D* and *S* as distances: distances the cell deviates from the uninjured state (analogous to how we measure temperature by liquid displacement over a distance in a thermometer). An ideal uninjured cell has no damage or induced stress responses (e.g., *D* = *S* = 0). Injuring it induces damage and stress responses in proportion to the injury magnitude. Specific individual damage and stress responses may be very high, others very low, but it is the aggregate of all that contributes to outcome. In general, tracking an individual damage product or stress response will more or less faithfully reflect the aggregate changes.

The empirical implication is that we do not actually need to measure every single damaged molecule and induced stress response in the cell to estimate *D* and *S*. We need markers that are: (1) representative of the general covariance, and (2) as inexpensive and reliable to measure as possible. For over half a century there has been what may be called the “relentless dissection” of the ischemic brain. This huge effort has revealed many, many of the pieces of wreckage ischemia induces, and has revealed the main players in the stress responses. From this substantial list, we can find markers of the general covariance and use these as means to ***estimate****D* and *S* without literally measuring every single damaged molecule and induced stress response.

There are now technologies that provide the means for measuring these deviations from the uninjured state: the -omics technologies. A decade ago, the line of thought I am proposing would have been impossible to implement in practice. Now, because of the -omics technologies, it is imminently practical.

### 6.2. Measuring *D*

To measure *D* we must track global changes in damage products. We could measure wholesale the lipid breakdown products using lipidomics. We could use proteomics to measure total amounts of protein aggregates or degraded proteins [35]. We could use metabolomics to measure altered metabolic products [36]. In all of these cases, the specific things we measure are less important than measuring *how much they change* from the uninjured condition. By definition, an uninjured cell has only some basal low level of damage. Thus, when the cell gets injured, there is increased concentration of a variety of different damage products over the basal background. If the cell recovers, the concentrations of damage products decay to zero. If the cell dies, the damage products also decay to zero, now because the cell no longer physically exists.

Initial pilot work could establish how different damage products covary. From this, pick the most representative that is also the most inexpensive and reliable to measure, and we have a means to measure *D*.

### 6.3. Measuring *S*

One of the most important, and general, aspects of the post-ischemic stress responses is they induce changes in gene expression. Many of the induced mRNAs code for stress response proteins [37]. With current microarray technology it is possible to measure all genes in the genome of some organisms. Thus, one can measure how far an injured cell moves in **gene space** away from the control condition. We now briefly described a pilot study to measure the genetic distance of microdissected CA1 and CA3 at 8 h reperfusion after 10 min global cerebral ischemia (8hR) compared to nonischemic, sham-operated controls (NIC).

It is now well-known that transcription does not necessarily correlate with translation [38]. However, translation is the functional endpoint of a gene product. *S* represents the *functional amount* of stress responses. Therefore, we reasoned that to estimate *S* we needed to consider not the total transcriptional changes, but the amount of new transcripts actually being translated. A means to detect translating mRNAs is to isolate polysomes and identify the associated mRNAs [39].

**Figure 10:**
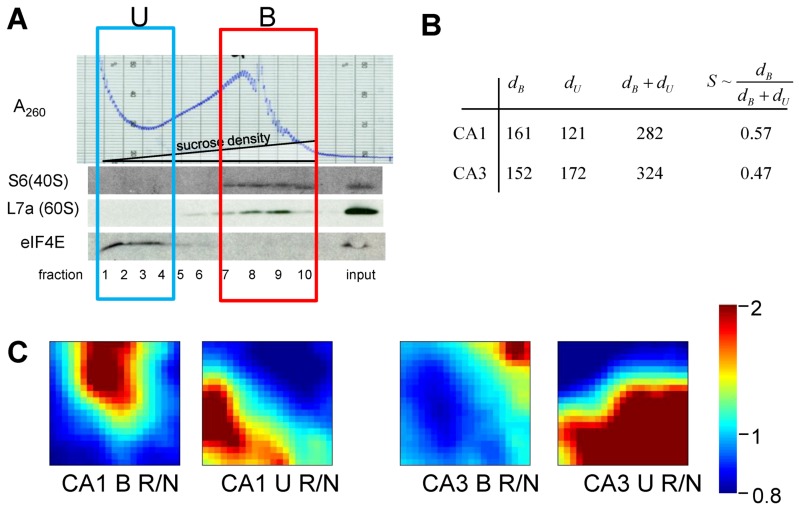
(**A**) Polysome profile for nonischemic, sham-operated controls (NIC) CA1. Westerns of fractions for ribosomal proteins S6 and L7a, marking 40S and 60S subunits, respectively. eIF4E is translation initiation factor 4E. Boxes mark polysome-bound (B) and unbound (U) fractions. (**B**) Gene distances for CA1 and CA3 polysome bound (*d*_B_) and polysome-unbound (*d*_U_) fractions. (**C**) gene expression dynamics inspector (GEDI) analysis of microarray 8hR/NIC (R/N) class comparisons. *S* ~ *d*_B_/(*d*_B_ *+* *d*_U_).

Our experimental groups were microdissected hippocampal CA1 and CA3 at 8hR in rat, and in NICs. There were *n* = 15 animals in each group. Homogenates from 5 animals per group were randomly pooled to give 3 replicates per group. Pooling was necessary to provide enough material to prepare polysome profiles by gradient centrifugation. RNA was extracted from: (1) polysome-containing fractions (polysome-bound mRNAs, B), and (2) the light gradient fractions that did not contain polysomes (polysome unbound, U) (Figure 10A). Next, 24 microarrays were performed for 8 experimental groups (*n* = 3 microarrays/group): NIC CA1B, NIC CA1U, NIC CA3B, NIC CA3U, 8hR CA1B, 8hR CA1U, 8hR CA3B, and 8hR CA3U.

Extracted RNA was hybridized to Rat Gene 1.0 ST microarrays (Affymetrix). Probe intensities were normalize (PLEIR/quantile) across microarrays, and averaged for each of the eight experimental groups. Next, class comparisons of CA1B 8hR/NIC, CA1U 8hR/NIC, CA3B 8hR/NIC and CA3U 8hr/NIC were calculated, resulting in four lists of gene ratios of for all 26,000 genes in the rat genome. Our only filter was that a given probe set had an Entrez ID, thereby eliminating ~9k probe sets, and leaving ~17k positively identifiable species that were subsequently aggregated. To estimate *S* we used Equation (9) as an intermediary formula:

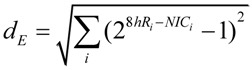
(9)

Equation 9 calculates the multi-dimensional Euclidean distance (the square root of the sum of the squares) that all genes deviated from the control condition. First the ratio of each gene *i* at 8hR compared to NIC is calculated by the standard formula 2*^8hRi-NICi^*, where *8hR_i_* and *NIC_i_* are the averaged, normalized intensities for gene *i* from replicate microarrays. If gene *i* does not change across conditions, this ratio equals 1. An unchanging gene contributes zero to the total distance, therefore, 1 is subtracted from the ratio. Then, the squares are summed over all genes and the square root taken to give Euclidean distance. In standard microarray analysis, statistical cutoffs eliminate genes below some X-fold ratio. A similar, though not identical, effect is achieved here because genes whose ratio approach 1 contribute progressively less to the distance calculated by Equation (9).

The results of treating the 8hR/NIC ratios by Equation (9) are shown in Figure 5B, where *d_B_* is the total distance traversed in polysome-bound mRNAs, and *d*_U_ is the total distance traversed by polysome-unbound mRNAs. For CA1 and CA3, *d*_B_ was 161 and 152, and *d*_U_ was 121 and 172, respectively. The total change in all transcription is estimated by adding *d_B_* and *d*_U_, giving 282 and 324 for CA1 and CA3, respectively. These numbers can be interpreted as follows. The distance polysome bound mRNAs changed between the regions was similar (161 *vs.* 152), but the overall change in transcription (282 *vs.* 324) was ~15% greater in CA3 compared to CA1. 

If we then take the ratio of polysome-bound to total transcriptional distances, we obtain a potential estimate of the variable *S*. This ratio represents the percentage of new transcripts that are polysome-bound, and presumably in the process of being translated. For CA1 57% of new transcripts are polysome bound, and in CA3, 47% are polysome bound, an approximately 20% difference. Thus, if we consider only total changes in transcription, here represented by *d*_B_ + *d*_U_, we might conclude CA3 under goes a greater stress response. But if we consider polysome-bound mRNAs as a fraction of all new transcripts, then the numbers suggest a ~20% greater stress response in CA1.

To complement the numerical results, the gene expression dynamics inspector (GEDI) method was used to visualize the same data that went into the numerical calculations (Figure 10C). GEDI organizes gene expression data using self-organizing maps, producing mosaic images, where each tile contains the same genes amongst all the samples, allowing visualization of the qualitative patterns in gene expression [40]. GEDI analysis revealed that the pattern of polysome-bound and -unbound transcripts were completely different between CA1 and CA3, relative to their respective control states.

There are weaknesses with this preliminary analysis. First, it assumes all new transcripts are protective and positively contribute to *S*. This may not be true, and the assumption is used only as a first approximation. Second, the issue of how to statistically analyze such data is unclear and so comparing the numbers is tenuous at best. Microarray statistical calculations are complex to account for correlations in gene changes, and the usual statistical methods (*t*-test, ANOVA, *etc.*) do not apply. Third, translation is known to be inhibited in CA1 pyramidal neurons at this time point [41], so to detect that 57% of all new mRNAs are polysome-bound in CA1 is a surprising result. The experimental design measured polysomes in all cell types in CA1 and CA3, so other cell types clearly contribute to the polysome-bound fractions, and this estimate of *S* is thus a weighted average of all cell types for each region. Finally, this single measurement is only one time point of one time course in an injury course. To be meaningful in the scope of the dynamical model, the *S* estimate needs to be plotted as a function of reperfusion to give *S* time courses for CA1 and CA3 at *I* = 10 min of 0% CBF. 

In spite of these very serious limitations, this pilot study opens up a new and different way to look at the biological data following ischemic injury and uses analytical methods that ultimately will allow empirically testing the nonlinear dynamical model. While the above is certainly the first attempt to quantify *S* after brain ischemia, it is not the first time this general approach has been used. Huang and colleagues were the first to use multidimensional distances in a gene network state space to measure cell differentiation [42], and their methods can be adapted to measuring cell injury as is illustrated here. 

To summarize this section, we can apply -omics technology to estimate *D* and *S* by measuring hundreds or thousands of biomolecules simultaneously, find a means to aggregate the result (e.g., Equation 9) and thereby measure *how much D* and *S* deviate from the control condition.

## 7. The Focal Ischemia Problem

We now come to the final major topic of the paper: what it would mean to have the fully determined solution to the model in hand. We illustrate this in two ways. In this section, we discuss how the master bifurcation diagrams allow us to model stroke or focal brain ischemia. In Section 8, we use the master bifurcation diagrams to understand neuroprotection. 

### 7.1. Other Cell Types in the Brain

To apply the model to focal ischemia, we need to know the values of *c_S_* and *λ_S_* for the other cell types in the brain. Figure 9 shows the master bifurcation diagrams for only how one type of neuron (a hypothetical CA1 neuron in this case) will respond to all possible levels and durations of CBF. Clearly we need to have equivalent diagrams for all cell types of the brain, because all cell types are affected by global ischemia and can potentially be affected by focal ischemia. Measuring other cell type’s injury dynamics will require a design like Figure 5. However, this clearly gives rise to technical obstacles that need to be overcome to determine parameter values of the individual brain cell types. Microdissecting CA1 approximates pure CA1 pyramidal neurons, but is still contaminated by interneurons, glia, and vascular elements. But for some brain regions, gross dissection is out of the question. For example, cerebral cortex cannot be dissected and homogenized to measure *D* or *S* for any specific type of cortical neuron; cerebral cortex is simply too heterogeneous and the in vitro methods described above to estimate *D* and *S* would be useless. To circumvent these issues, one approach might be to use markers of *D* and *S* that can be visualized by the microscope. Laser capture also offers a means to isolate single cell types. This is a formidable technical barrier, but one that is not, in principle, insurmountable. To continue the discussion, we assume this problem has a solution.

### 7.2. Parameter Values between Global and Focal Ischemia

Assuming we can overcome the technical hurdles and get the *c_S_* and *λ_S_* parameter values for all cell types in the brain, we then must face the assumption that the parameters we measured in the global model carry over to focal ischemia. It seems intuitively apparent that *c_S_* and *λ_S_* should be the same irrespective of the mode of injury since they measure intrinsic features of a given cell type. That is to say, *c_S_* and *λ_S_* measure the intrinsic strength of a cell type’s stress responses, which are genetically endowed. Thus, *c_S_* and *λ_S_*, should be constant for a given cell type across different insults. 

It is less clear if *c_D_* and *λ_D_* will be the same between global and focal models. Given the complex and interlocked responses between neurons, glia, vascular elements and the immune system, under the heterogeneous blood flow conditions of focal ischemia [43,44], it is possible that *c_D_* and *λ_D_* might be different between global and focal ischemia. On the other hand it is hard to imagine they would vary radically from parameters obtained from global models.

Existing empirical evidence is already quite clear that the main regional responses of the brain in focal ischemia mimic the features of different conditions of global ischemia. Briefly summarizing what we discussed before [13]: The necrotic core is similar to the necrosis that neurons undergo after long durations of complete global brain ischemia (e.g., >30 min of 0% CBF). Penumbral neurons have much in common with the delayed neuronal death phenotype that CA1 neurons display at 10 min ischemia [45,46]. The area of benign oligemia in stroked brains [47] has overlapping features with nonlethal preconditioned neurons after global ischemia [48,49]. This phenomenological data argues that we should expect the values of *c_D_* and *λ_D_* between global and focal ischemia to be, if not identical, then at least similar. 

### 7.3. Applying Global Ischemia Master Bifurcation Diagrams to Focal Ischemia

**Figure 11:**
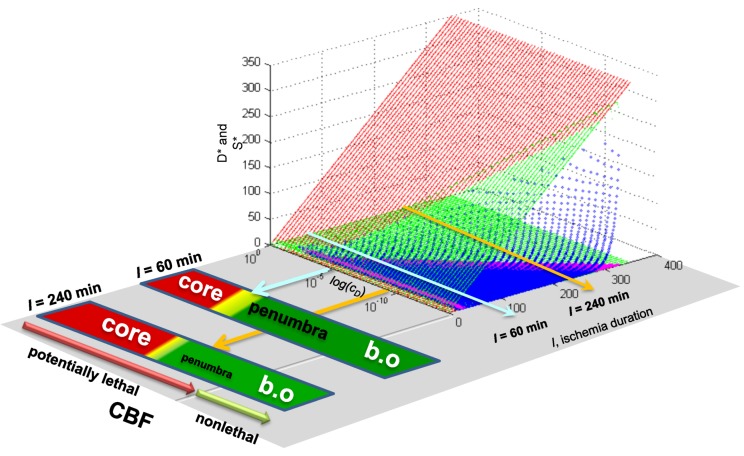
Outcome across the *c_D_*/CBF continuum at two different values of *I*. Light blue arrow on master bifurcation diagram indicates *I* = 60 min and corresponds to outcome plot labeled *I* = 60 min. Orange arrow on master bifurcation diagram indicates *I* = 240 min and corresponds to outcome plot labeled *I* = 240 min. Master bifurcation diagram applies only to the lethal CBF range of 0% ≤ CBF ≤ 40% as calculated by Equation (6). “Core” and “penumbra” are as commonly understood in the stroke literature, and b.o. is benign oligemia.

The simulated, fully determined global ischemia, allows us to see how the correspondence between focal and global ischemia works out in practice. Figure 11 shows co-plots of the D* and S* master bifurcation diagrams for global ischemia, taken from Figure 9, next to which is placed the scale of CBF, since it forms a direct relationship with *c_D_* (Figure 9E, orange line). This image allows us to determine outcome along the entire CBF axis, given some *I* value. In the present contest, *I*, the duration of focal ischemia, is meant to model a permanent focal ischemia. For such a given *I*, some cells will recover and others will die. At *I* = 60, cell death occurs at *c_D_* > ~10^−5^, corresponding to CBF ~ 12%, as calculated from Equation (6); for all CBF > 12%, the cells eventually recover. However, for *I* = 240, cell death now occurs at *c_D_* > ~10^−9^, corresponding to CBF ~ 25%.

When outcome is plotted next to the CBF scale, we get three phenotypes: (1) cells that die, (2) cells that run through injury time courses but recover, and (3) unaffected cells whose CBF > 47%. These are readily associated with core, penumbra and benign oligemia, respectively. As seen in Figure 11, the longer duration (*I* = 240) gives a larger range of CBF corresponding to a core region, and corresponding decrease in penumbra-like response. Of course, in a real focal ischemia we do not expect a continuum of CBF values because of vascular anatomy.

This example shows that, for a given *I* (e.g., duration of focal ischemia), there will be simultaneously different values of *c_D_* in the same brain, corresponding to the different CBF levels. Thus, in this fashion, solving global ischemia lays the foundation for modeling focal ischemia. If the parameters values are similar between global and focal ischemia, then by solving global ischemia, we implicitly solve focal ischemia.

### 7.4. Spatial Considerations for Focal Ischemia

We must confront a new dimension to the focal problem that is not intrinsically present in the global ischemia problem, and already mentioned above: space. The global ischemia problem is mainly one of time. The same temporal considerations occur during focal ischemia, but the spatial distribution of blood flow gradients adds a new dimension to the problem.

From a computational perspective, this issue is manageable. For example, it is possible to generate a 3D lattice where each vertex represents a cell in the brain. A specific CBF gradient is superimposed over this lattice. Then, the cell injury model is run in parallel at each vertex, where *c_D_* is a function of the spatial distribution of the CBF gradient. The CBF gradient could be modeled as realistically as possible, up to simulating the entire brain vascular anatomy and physiology. The answer to this math simulation would tell not only which points in the lattice live and which die, given some spatial blood flow gradient, but the time courses of recovery or death for each point in the lattice. It would take some computational power, but that is getting cheaper all the time.

What kind of payoff can we potentially expect from such efforts? Real time stroke simulations could be of great utility in a clinical context. With noninvasive neuroimaging indicating the anatomy and CBF levels of a given stroke, simulations could, in principle, be run at the bedside to prognosticate outcome on a patient-by-patient basis; a long sought goal for neurologists [50,51].

Thus, in broad outline at least, the focal problem is in within reach. Clearly serious technical hurdles and computational issues exist that must be overcome to convert theory into practice. But the program we are suggesting here outlines a clear path and direction achievable with focused effort.

## 8. Neuroprotection from a Nonlinear Perspective

### 8.1. Preliminary Thoughts: Neuroprotection Is Technology Not Science

We come now to the final main topic of this paper. We now use the hypothetical results obtained above as a platform to study neuroprotection. Neuroprotection is currently thought of in terms of inhibiting some specific change in post-ischemic neurons, call it “x”, where x is a molecular pathway, physiological, or pathophysiological process claimed to cause cell death. But this logic has not successfully translated to the clinic. The two most neuroprotective manipulations known—preconditioning and hypothermia—do not possess a clear-cut “inhibit x” logic, and both have, like ischemia itself, eluded descriptions in terms of linear molecular pathways and cascades [52]. However, each treatment alters damage accumulation and stress response activation. Our model offers the alternative interpretation that these technologies are effective because they alter cellular injury dynamics.

Clearly, if achieved, neuroprotection will represent a successful technology. Scientific success translates into effective technology. Some examples of important technological advances and the corresponding science include: classical Newtonian dynamics put men on the Moon and the Curiosity on Mars. Maxwell’s electrodynamics gave us radios and telephones. Quantum mechanics gives us computers, cell phones and MRI instruments, among other marvels. Thermodynamics gives us engines, heaters and refrigerators. Each of these technologically successful sciences utilizes dynamics. What does dynamics bring to the table that offers such success?

Let us consider one example. Classical Newtonian mechanics recently put Curiosity on Mars. But what is classical Newtonian mechanics? It involves the dynamical equations describing how matter moves under the influence of gravity. Does Newtonian mechanics tell us what matter *is*? No. Does it tell us what gravity is? No. So, the two main aspects of Newtonian mechanics, matter and gravity, are unexplained by the theory. Yet, putting Curiosity on Mars is widely considered a success. The success stems from physicists recognizing that phenomena occur on different scales. The nature of mass and gravity are problems at a micro-scale compared to characterizing the behavior of macroscopic objects moving in gravity field, which is the domain of Newton’s mechanics. It is a “divide and conquer” strategy that has worked well: solve what you can, and work towards solving what you cannot. Thus, classical mechanics tells us *how* macroscopic objects behave in gravity without knowing precisely *what* causes them to do so.

Bringing this discussion back to brain ischemia, we suggest it will not be knowledge of *what* ischemia does at a cellular or intracellular level that will give us neuroprotection, but knowledge of *how* ischemia causes cells to change over time. From a functional (*i.e.*, clinical) point of view, the most important change is whether the cell recovers or dies. Thus, the practical information we seek is to know *how* ischemia either does or does not kill cells as a function of time. This is captured in the dynamical Equation (1), which, similar to the gravity example, provides a foundation to manipulate trajectories for technological purpose, where the technology in the present case is neuroprotection.

### 8.2. A Precursor of the Nonlinear Dynamical Model

As a stepping stone to discussing neuroprotection in the context of the nonlinear cell injury model, we discuss work from the laboratory of J.A. Zivin (we thank Dr. Bingren Hu for bringing this work to our attention, [35]). This work can be interpreted as a precursor of the nonlinear cell injury model, and the empirical results obtained provide a template of how neuroprotection studies need occur in the context of the nonlinear cell injury model.

In their 1994 paper [53], Aronowski *et al.*, present a “graded bioassay” of focal ischemia using the 4 parameter logistic nonlinear regression model:

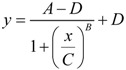
(10)
They interpreted the parameters as:

*y* = infarct volume,
*x* = duration of ischemia,
*A* = response at time zero (effectively, a background measurement),
*B* = “steepness factor” (effectively, the Hill coefficient),
*C* = ischemia duration giving a 50% infarct volume (*i.e.*, a 50% threshold, analogous to that in Equation 6),
*D* = maximum infarct volume.

Equation (10) is one of the many regression forms used in pharmacology to fit empirical data [54], and shares similar logic to our cell injury model. Retaining the parameter names, Equation (10) is similar to the following expression:

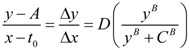
(11)

Equation (11) restates Equation (10) as a rate expression in the classic Hill equation form. Equation (11) can be interpreted to say: the rate of change of infarct volume (Δ*y = y − A*) with respect to the rate of change of ischemia duration (Δ*x =* ischemia duration *−* time zero) equals the maximum infarct volume (*D*) times a Hill function of the infarct volume (*y*) and 50% threshold for forming an infarct volume (*C*), taken to the Hill coefficient, *B*. Reinterpreting Equation (10) in this form illustrates the similarity to the logic to Equation (1).

**Figure 12:**
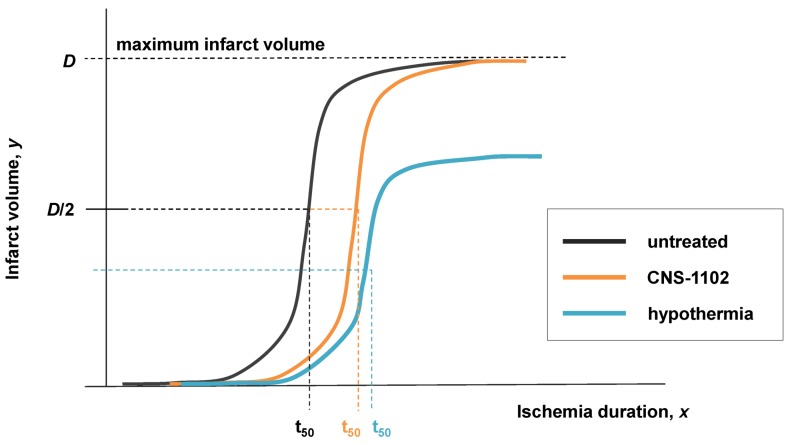
Schematic reproduction of data in Aronowski *et al.* (1994) [53]. Axes as indicated, and color-coded curves as indicated and explained in the text.

However, the important difference is that Equation (10) is an algebraic expression used to regression fit data, and has no underlying theory. Equation (1) is a differential equation that constitutes a theoretical stance on the cause of outcome following cell injury, e.g., that outcome is caused by an exponentially-driven competition between total damage, *D*, and total induced stress responses, *S*. Thus, it is not possible to directly covert Equation (10) to Equation (1), and the variables and parameters of Equation (10) are valid in only the limited circumstance of quantifying how infarct volume changes as a function of ischemia duration.

In spite of the theoretical limitations of Equation (10), Aronowski and colleagues put it to important empirical use demonstrating the effects of neuroprotective manipulations on infarct volume outcome. Their 1994 paper showed a very important result. In Figure 2 of Aronowski *et al.* (1994) [53] the effect of hypothermia and the effect of the NMDA receptor antagonist CNS-1102 on infarct volume are measured in terms of Equation (10). They show that CNS-1102 increases *C*, the 50% threshold ischemia duration, from 45 min in controls to 66 min in drug-treated. Hypothermia similarly increased *C* from 45 to 69 min, but also decreased, *D*, the maximum infarct volume, approximately 33% from the untreated condition. A reproduction of their data is shown in Figure 12.

The interpretation of this data is straightforward: CNS-112 only slowed the onset of cell death, but hypothermia not only slowed but also decreased the amount of cell death. 

We now show how the empirical results obtained by Aronowski and colleagues can be seen to be a special case of the nonlinear cell injury model, and can be interpreted as measuring and manipulating stroke injury dynamics. The results in [53] may be interpreted as empirical support for the general approach described here and bodes well for its future success.

### 8.3. Neuroprotection and Nonlinear Cell Injury Dynamics

Our previous work [12] laid a foundation for applying the cell injury model to neuroprotection. To briefly summarize the main result: a bistable phase plane contains attractors for both death and recovery. Thus, in bistable phase planes where *I* > *I_x_*, there is, in principle, the possibility of reversing the injury by shifting the cell from a pro-death to a pro-recovery trajectory. We called this “flipping state”. In a lethal monostable phase plane, there is only a pro-death attractor; the system will deterministically die, but there remains the possibility of slowing the progression to cell death by shifting the system to a trajectory with a longer duration.

In the scope of the present model, other trajectories on a phase plane are accessed by altering the initial conditions (*D*_0_, *S*_0_) [12]. This corresponds to a pre-treatment. The issue of modeling a post-injury treatment is broached in the final section. Starting from initial conditions, (*D*_0_, *S*_0_) = (0, 0) means starting from the uninjured state where both *D* and *S* are initially zero. We interpret altered initial conditions as pre-treatments that do the following [12]: (1) inhibit damage (*D*_0_ < 0), (2) increase damage (*D*_0_ > 0), (3) inhibit stress responses (*S*_0_ < 0) or, (4) activate stress responses (*S*_0_ > 0). In general, protection is achieved by inhibiting damage and/or activating stress response. As seen, inhibiting damage by pre-treatment introduces negative values for the variable *D*. 

The maximum protective effect is achieved, at least in principle, by pre-inhibiting 100% of the damage formed or by pre-activating the stress responses to 100% of their maximum value. In the normalized form, Equation (9), these correspond to *D*_0_ = −1 and *S*_0_ = 1, respectively. That is to say, no neuroprotection can function by inhibiting more than 100% of the possible amount of total damage, *D*, that could form in the system, nor activate total induced stress responses beyond 100% of their capacity to be activated. 

This is a critical insight. At the beginning of the paper it was stated that the model can describe all possible states of neuroprotection in the abstract, independent of the physical instantiation of any specific form of neuroprotection. On the landscape of current thoughts about neuroprotection, this is an alien idea. But it follows merely from using a deductive method, and is a familiar mode of thinking to workers in physical sciences and engineering. In what follows, we illustrate this mode of thinking, in part as a didactic exercise, but also as a substantive task to demonstrate the deduction of important theoretical consequences of applying the cell injury model to neuroprotection.

### 8.4. How to Measure a Presumed Neuroprotectant

The procedure for measuring the effectiveness of a neuroprotectant is similar to that used by Aronowski and colleagues [53]. However, their endpoint was infarct volume, a gross measure of outcome. Our endpoint measures are always *D* and *S* time courses. From these, the D* and S* bifurcation diagrams are obtained, which contain information not only about outcome, but also allow calculation of the trajectories to recovery or death. 

In brief, to measure a neuroprotectant, one must repeat a design like that in Figure 5 in the presence of the presumed neuroprotectant and determine if the bifurcation diagram changes, and if it does, to what extent. We illustrate this process below. Thus, there is no “short cut” to determining the efficacy of an ostensible neuroprotectant. By now it is clear that there is no magic “silver bullet” cure for brain ischemia. Any candidate neuroprotectant will have to be assessed via time series designs like those of [53] or as shown in Figure 5. We now show that, given a fully determined system, one can know beforehand the maximum neuroprotection under all possible injury circumstances.

### 8.5. Neuroprotection across an Injury Applies to Global Brain Ischemia

In this section we study neuroprotection at one specific CBF decrement, simulating neuroprotection following a global ischemic insult. We study the effect of altering initial conditions, in three ways: (1) maximally inhibiting damage, (2) maximally activating stress responses, and (3) both maximally inhibiting damage and maximally activating stress responses. We shall find that each case is different. 

We return to the results of the mock simulation and evaluate these for neuroprotective capacity. In Figure 8C, the purple rectangles mark the bistable regions of the bifurcation diagrams and *I_x_* is indicated by vertical lines. The dashed arrows from *I_x_* to the right end of the bistable region mark the **therapeutic region** of each bistable injury course. The therapeutic region of an injury course is the range of lethal values of *I* that are bistable and hence, could in principle flip state [12]. In our simulated 0% CBF injury course, there is no bistability and therefore no possibility to flip state. However, as CBF increases, the therapeutic regions increase considerably (Table 4), indicating the possibility for significant salvage at the higher CBF rates. 

**Table 4:** Therapeutic Regions (TR) for the 0%, 10%, 25% and 40% CBF cases. *I_b,max_* is the maximum bistable value of *I* in that injury course. TR = *I_b,max_* − *I_x_*.

CBF (%)	*I_x_*	*I_b,max_*	TR
**0**	10.0	10.0	0
**10**	26.6	53.8	27.2
**25**	51.3	187.2	135.9
**40**	74.2	318.6	244.4

**Figure 13:**
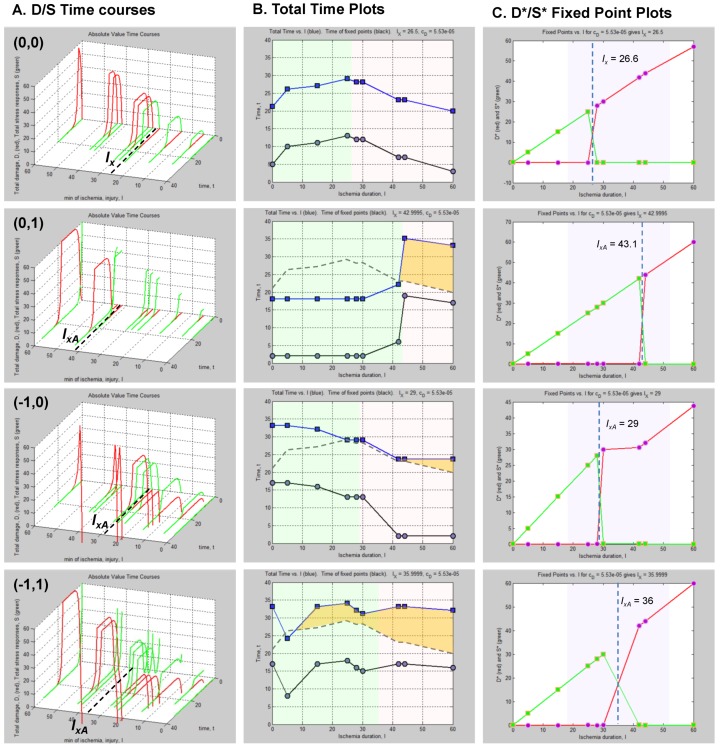
Simulations showing effect of neuroprotective pre-treatments on the 10% CBF injury course. Rows 1–4 are initial conditions (*D*_0_, *S*_0_) of (0, 0), (0, 1), (−1, 0) and (−1, 1), respectively. (**A**) *D* (red) and *S* (green) time courses under specified initial conditions. (**B**) Total time plots: total durations (blue) and attractor state durations (black) *vs. I*. The total duration from the (*D*_0_, *S*_0_) = (0, 0) are reproduced as dashed gray line on other plots. Orange area marks difference between the current and (*D*_0_, *S*_0_) = (0, 0) total duration curves. Green areas correspond to survival time courses, and red areas to death time courses. (**C**) Fixed point plots. Purple rectangle marks full extent of the bistable region, which does not change under altered initial conditions. *I_x_* is the cross-over amount of *I*, calculated by Equation (5). *I_xA_* is the “apparent *I_x_*” and is where the D* and S* fixed point curves cross when (*D*_0_, *S*_0_) ≠ (0, 0).

In this section, we illustrate the effects of neuroprotective pre-treatment manipulations on the 10% CBF case. Row 1 in Figure 13 repeats the 10% CBF simulation starting from initial conditions (*D*_0_, *S*_0_) = (0, 0). Row 2 starts from initial conditions (0, 1), representing 100% pre-activation of stress responses. Row 3 starts from initial conditions (−1, 0), representing 100% pre-inhibition of damage. Row 4 starts from initial conditions (−1, 1), representing both 100% pre-inhibition of damage and 100% stress response pre-activation. Columns 1, 2 and 3 of Figure 13 show, respectively, the *D* and *S* time courses, total duration plots, and the fixed point plots, for each case.

Two main results are obtained from these simulations. First, the injury dynamics are differentially altered by the pre-treatments; the *D* and *S* time courses are altered (column 1) as are the total durations of recovery or death (column 2). Second, each pre-treatment has a different effect on outcome.

#### 8.5.1. Injury Dynamics

The changes to the forms of the *D* and *S* time courses, and their altered durations, follow from the fact these are trajectories different from those accessed when initial conditions are (0, 0). By starting *S* from 1, it will take longer for damage to inhibit the stress responses in lethal phase planes. Starting *D* from −1, it will take longer for the system to build up enough damage to inhibit the stress responses. Therefore all three manipulations had the effect of lengthening the time to death in lethal bistable phase planes, although they did so to different extents. The increased duration in the time to death is indicated by the orange areas on the total time plots (Figure 13B). We thereby reproduce one of the two results shown in the Aronowski *et al.* paper [53].

#### 8.5.2. Outcome

Perhaps surprisingly from an intuitive point of view, each manipulation had a different effect on outcome, which can be characterized by the shift in the apparent cell death threshold, designated as *I_xA_* on the Figure 13 plots. *I_xA_*, the **apparentvalue of *I_x_***, is that *I* value where D* = S* when the system does not start from (0, 0), readily determined by the crossing of the D* vs. I and S* vs. I curves. *I_xA_* is not *I_x_*. *I_x_* is calculated by Equation (5), and remains unchanged no matter what the initial conditions. *I_xA_* instead represents how far into the therapeutic region the altered initial conditions were able to cause the system to flip state, which we now quantify.

For each of the initial conditions, (0, 1), (−1, 0) and (−1, 1), *I_xA_* was 43.1, 29 and 36, respectively. Subtracting *I_x_* from *I_xA_* for each manipulation gives 16.5, 2.4, and 9.4, respectively. The therapeutic region (TR) of our hypothetical 10% CBF is 27.2 *I* units (Table 4). The **penetration into the therapeutic region** is calculated by the formula (*I_xA_* − *I_x_*)/TR × 100%. Therefore the (0, 1), (−1, 0) and (−1, 1) manipulations extended, respectively, 61%, 9% and 35% into the therapeutic region of this injury course.

This result is interpreted as follows. With no prior manipulation (e.g., initial conditions equal (0, 0)), the system dies at 26.6 min or greater of 10% CBF. If stress responses are 100% pre-activated, the system now dies at >43.1 min of 10% CBF, extending an additional 16.5 min past *I_x_*. For 100% inhibition of damage, the system dies at >29 min (2.4 min past *I_x_*). If there is both 100% inhibition of damage and 100% pre-activation of stress responses, the system dies at >36 min of 10% CBF (extending 9.4 min past *I_x_*). There are three important features to emerge from this example.

First, **inhibiting damage is not equivalent to activating stress responses**. Clearly, in this example, this effect is not biological but is mathematical. It is the particular structure of the phase planes that will determine the effects of changing initial conditions. The structure of the phase planes in turn is determined by the parameter values used in Equation (1). For our hypothetical system, the value of *c_S_* is much greater than that of *c_D_* (*c_S_* >> *c_D_*). This generates an asymmetrical phase plane such that changing *S*_0_ has greater access to the survival attractor than changing *D*_0_. However, while this is a purely mathematical consideration, it alerts us to the possibility that a similar dynamic may play out biologically.

Second, **the effect of altering *D*_0_ and *S*_0_ was not additive**. The (−1, 1) manipulation was not the sum of the (−1, 0) and (0, 1) manipulations. This effect is also due to the structure of the phase planes. The non-additive nature of the manipulations reinforces that we are dealing with a nonlinear system where emergent behaviors cannot be predicted beforehand, and can results in counter-intuitive effects.

Third, and of particular significance in the context of neuroprotection, **the system could not be made to flip state across 100% of the bistable region**. This too is a consequence of the phase plane structure. Although both a pro-survival and pro-death attractor are present on a given bistable phase plane, they may not be accessible from a specific initial condition, and that is the case here. Physically, it makes no sense to imagine greater than 100% activation of stress responses or greater than 100% inhibition of damage, which confines initial conditions to the range 0%–100%. In the present example, the (0, 1) case extended farthest into the therapeutic region, accessing 61% of it. To access the pro-survival attractors at *I* > 43.1 requires initial conditions outside the range of [0, 1] for either *D* or *S*. Thus, the bistability exists mathematically, but not biologically, and protection, in principle, cannot be achieved across the entire therapeutic region.

We showed previously [12] a system can only flip state in a lethal bistable phase plane; for any monostable phase plane where *I* > *I_x_*, survival is impossible. We now demonstrate here a 2nd intrinsic limit on protection: even given a range of bistable phase planes with *I* > *I_x_*, there is the possibility that survival attractors will not be accessible across the entire therapeutic region, as illustrated by the present example. In the next section, we will discover a third dynamical limit to any attempt at neuroprotection: the benefit garnered with increasing initial conditions is a case of diminishing returns.

### 8.6. Neuroprotection across Multiple Injury Courses

We now come to the final section of this paper. The previous section dealt with neuroprotection across a single injury course. Such thinking would be appropriate in the context of global brain ischemia, where, in our present hypothetical system, a single level of CBF corresponds to a single value of *c_D_*. In this section, we study what protection would look like across multiple injury courses, corresponding to multiple levels of CBF, and move towards a situation resembling focal ischemia. Our goal is to calculate the maximum ability to “flip state” given the master bifurcation diagrams of our system.

Setting up and solving the problem is identical to the previous section, except now we do so for several injury courses simultaneously. For this example, we will use the following values of % CBF to define our injury systems: 0%, 2.5%, 5%, 10%, 25%, and 40%. Two additional CBF levels are added (2.5% and 5%) to help illustrate graded effects that emerge from this example. Since, in our hypothetical system, manipulating only *S*_0_ gave a maximal effect over either manipulating only *D*_0_ or manipulating *D*_0_ and *S*_0_ in combination, we only consider changing *S*_0_ while keeping *D*_0_ = 0.

We first consider the maximum possible salvage at *S*_0_ = 1 (100% total stress response activation). As seen in Table 5, the two lowest CBF levels, 2.5% and 5% penetrated fully across the therapeutic region, which is to say, at 2.5% and 5% CBF, each could flip state across 100% of the therapeutic region. However, with increasing %CBF, the penetration into the therapeutic region decreased: For 10%, 25% and 40% CBF, the penetration was 77%, 43% and 50%, respectively. 

**Table 5:** Maximum penetration into therapeutic regions for six CBF levels when injury courses are run at *S*_0_ = 1.

	0% CBF	2.5% CBF	5% CBF	10% CBF	25% CBF	40% CBF
*I_xA_* − *I_x_*	0	0.8	5.5	16.5	54.6	127.5
TR	0	0.8	5.5	21.4	130.7	253.4
(*I_xA_* − *I_x_*)*_x_*/TR %	0.0%	100%	100%	77%	43%	50%

**Figure 14:**
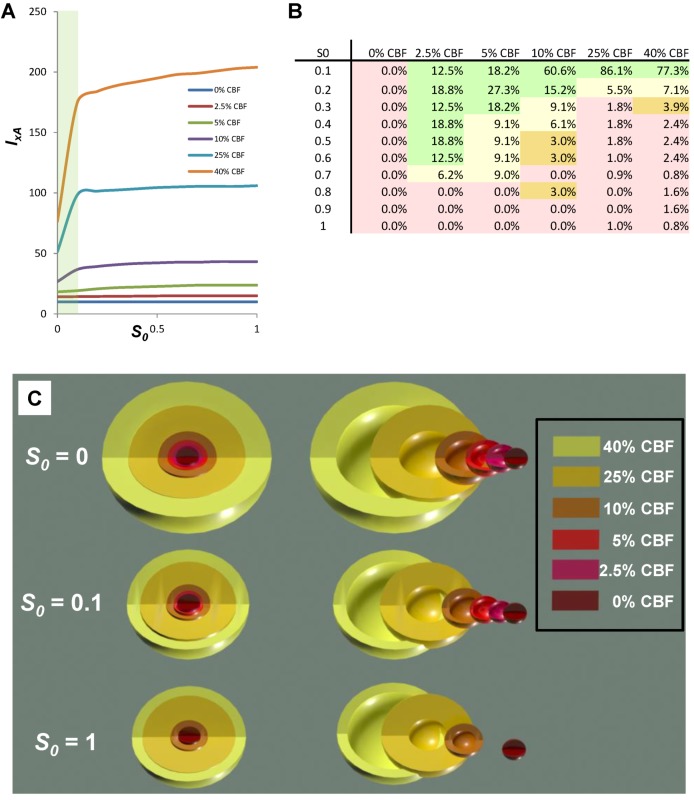
Altered initial conditions across multiple injury courses models stroke neuroprotection. (**A**) Plots of *I_xA_ vs. S*_0_ at various %CBF levels as indicated. Green area marks *S*_0_ ≥ 0.1. (**B**) Table showing change in *I_xA_* as a percent of the total change, with 0.1 increments in *S*_0_ at the %CBF levels indicated. Green, yellow, orange and red indicate large, medium, small and very small changes, respectively. (**C**) Simple geometrical model of nested concentric spherical shells provides a crude application of the nonlinear dynamical model to stroke injury and stroke neuroprotection. Key indicates correspondence between spheres and %CBF. Top row is untreated case, middle row is 10% of maximal protection (*S*_0_ = 0.1), and bottom row is maximal protection (*S*_0_ = 1).

We next ask how each injury course behaves across the range 0 ≤ *S*_0_ ≤ 1. This is interpreted as pre-activating stress responses to 10%, 20% and so on up to 100%. For each of the six CBF levels, each injury course is solved for 0 ≤ *S*_0_ ≤ 1, in 0.1 increments, and *I_xA_* calculated at each value of *S*_0_. The result of *I_xA_ vs. S*_0_ is plotted in Figure 14A. Each curve starts at its respective value of *I_x_* and ends at the maximum obtainable *I_xA_*. As CBF% increases, the curves rise more steeply, but then level out.

The Table in Figure 14B is color coded to show the increase in protection as *S*_0_ is stepped up every 0.1. The green, yellow and orange cells indicate large, moderate and small increases, respectively. The red cells indicate zero or trivially small changes. As seen, increasing *S*_0_ has progressively less effect as % CBF increases. This table is a wonderful example of emergence in a nonlinear system. Its interpretation is that only the 2.5% and 5% CBF levels merit the attempt to try to increase *S*_0_ beyond 0.1. For the 10%, 25% and 40% cases, the additional benefit of trying to increase *S*_0_ past 0.1 is very small, which is to say, it is a case of diminishing returns.

By calculating the effect of changing *S*_0_ for six discreet injury courses (*i.e.*, six values of *c_D_*), the next logical step would be to calculate it for the entire master bifurcation diagram across the continuum of *c_D_* values. We do not perform this calculation here. Instead, we conclude asking: can we use the information in Figure 14A,B to get some sense of how these magnitudes of protection would play out in a scenario resembling real ischemic brain injury? To answer this question, and as discussed above, we need a geometrical model that contains all six injury courses simultaneously. A very simple model that crudely approximates a blood flow gradient is to imagine six concentric spherical shells of decreasing radius, where each shell is associated with one of the CBF levels. The volume of each shell is taken to be is the spherical volume (4/3π*r*^3^) minus the volume of the next smaller sphere inside it, producing a series of shells of discreet CBF levels.

This geometry is shown in Figure 14C where each shell is labeled with its corresponding CBF level. Because each of the CBF levels is lethal, each shell represents the maximum extent of cell death at that CBF level as *I* goes to infinity. If the simulation is run from initial conditions (0, 0) the volume of each shell would represent dead cells, or “core” in stroke parlance.

Our simple calculation is to determine the volume of each shell that would **not** die given a neuroprotective treatment. We do this by simply multiplying the percent penetrations into the therapeutic region for each CBF level and multiple these by the shell volumes for each CBF level. For example, at 2.5% CBF, 100% of the volume would survive at *S*_0_ = 1. But for 40% CBF, only 50% of the volume survives at *S*_0_ = 1 (Table 5).

These results are graphically depicted in Figure 14C that compares the *S*_0_ = 0, *S*_0_ = 0.1 and *S*_0_ = 1 cases visually. The first row shows the total volumes that would die in the untreated state. The 2nd and 3rd rows show the volumes that would **not** die with treatments that pre-activate stress responses 10% and 100%, respectively.

We take the sum of all the shell volumes to be 100%. For *S*_0_ = 1, 49% of the total volume of the shells would not die. If we repeat the calculation at *S*_0_ = 0.1, then 38% of the total volume of the shells would not die; that is to say, there would be a 38% reduction in cell death.

This is an uncanny result. From purely theoretical considerations we have come close to the typical reduction in infarct volume of 25%–33%, as seen, for example with the effect of hypothermia on infarct volume in the study of Aronowski *et al.* (1994) [53] in Figure 12. Again, however, given the arbitrary nature of the parameter values underlying our hypothetical example, and the unrealistically simple CBF geometry, this may just be a fortuitous coincidence. Nonetheless, crude though this calculation may be, it illustrates in a simple way how the nonlinear dynamical model of cell injury can be applied to the problem of neuroprotection after focal ischemia.

This simplified and hypothetical example shows that 100% protection is impossible, even in principle, and that, in this specific example, there is a case of diminishing returns to achieve the maximum possible protection.

## 9. Conclusion

We close discussing two issues: (1) the possibility that Equation (1) is not the correct form for describing cell injury and (2) how to begin to think about post-treatments in the scope of the model.

### 9.1. What If Equation (1) Is Wrong?

The real crux of this issue is the status of the proposed variables *D* and *S*. We suggest that the concepts are quite intuitive. It is impossible to deny that any injury will cause some total amount of damage and some total induced stress response in the cell. The issue is: does monitoring their change in time reveal the injury dynamics? Our mathematical results, and the empirical work of Aronowski and colleagues [53], support that *D* and *S* will serve as operationally useful concepts.

Granting the utility of *D* and *S*, if the protocol outlined in Figure 5 is followed, it is inevitable that it will reveal the injury dynamics of global brain ischemia. Using the large variety of analytical methods of time series analysis available, from this empirical data it will be possible to discern the underlying dynamics. It is possible that, upon performing the experiments, Equation (1) is not the best expression to describe the dynamics. In that case, Equation (1) will have been a stepping stone to performing the experiments in the first place. But what is certain is that performing the experiments will reveal the dynamics. This paper has illustrated the benefits to be derived from having in hand the correct dynamical expression, fully determined by empirically-measured parameter values. Given what has been illustrated here, the imperative to figure out how to measure *D* and *S* and perform these experiments is now acute.

### 9.2. Post-Treatments

There is a startling revelation that follows from the logic used here. By conceptualizing cell injury in terms of total damage, *D*, and total stress responses, *S*, this implies that drugs act, not by targeting specific molecular pathways as is widely believed today, but by altering cell injury dynamics. This is an unexpected aspect of the cell injury model that has only become apparent in the course of working with the model. **The model itself is a theory of pharmacodynamics**. In this theory, drugs are just another damage agent that perturbs cell homeostasis. In short, all drugs are poisons. They exert their beneficial effects not by targeting “pathway x”, but by serving as low intensity damage agents that exert an essentially pre-conditioning-like response. Because most drug studies assay only a highly circumscribed aspect of the molecular response, it is possible that all drugs activate many molecular pathways, including stress responses, and these are simply missed because they are not assayed. It would be difficult to use existing evidence to argue against the idea that drugs widely influence cell molecular biology.

If indeed this proposition is true, that drug action in general is simply that of a low level damage agent, then the proper way to formulate neuroprotection would be to modify Equation (1) to accommodate more than one injury at a time. We have begun this project and call it the “multi-injury model,” which allows simultaneous or sequential application of damage agents in an injury system. This provides a means to model a post-injury treatment. The details of this model will be described in a future communication.

To close the present paper, we have illustrated by example how to execute an empirical program to solve the brain ischemia problem based on a nonlinear dynamical view of cell injury. We have illustrated a systematic means to formulate and measure brain ischemia that allows the maximum potential for any possibility of neuroprotection to be extracted and exploited technologically.
